# Identification and ultrasensitive photoelectrochemical detection of LncNR_040117: a biomarker of recurrent miscarriage and antiphospholipid antibody syndrome in platelet-derived microparticles

**DOI:** 10.1186/s12951-022-01608-1

**Published:** 2022-08-31

**Authors:** Zhiwei Sun, Qian Zhou, Yufei Yang, Lei Li, Mengru Yu, Hui Li, Aihua Li, Xietong Wang, Yanyan Jiang

**Affiliations:** 1 Key Laboratory for Liquid−Solid Structural Evolution and Processing of Materials, Ministry of Education, Shandong University, Jinan, 250061 China; 2 Department of Obstetrics and Gynecology, Shandong Provincial Hospital Affiliated to Shandong First Medical University, Jinan, 250021 China; 3 Department of Obstetrics and Gynecology, Liaocheng People’s Hospital, Liaocheng, 252000 China; 4 Key Laboratory of Birth Regulation and Control Technology of National Health and Family Planning Commission of China, Maternal Child Health Hospital of Shandong Province, Jinan, 250014 China

**Keywords:** Antiphospholipid antibody syndrome, Platelet-derived microparticles, LncRNAs, Photoelectrochemical biosensor

## Abstract

The abnormal expression of long non-coding RNAs (LncRNAs) in platelet-derived microparticles (PMPs) is closely related to immune disorders and may lead to antiphospholipid antibody syndrome and recurrent miscarriage. To understand the association between the LncRNAs in PMPs and RM/APS, the differences in the expression of LncRNAs in RM/APS patients and healthy controls were analyzed. Microarray analysis and RT-qPCR detection proved that RM/APS patient exhibited high levels of LncNR_040117 expression. The lentiviral silent expression transfection of HTR-8/SVneo cells indicated that LncNR_040117 downregulation decreased the activity of HTR-8/SVneo cells and inhibited the MAPK signaling pathway, further confirming the biomarker proficiency of LncNR_040117 for RM/APS. After that, we proposed a β-In_2_S_3_@g-C_3_N_4_ nanoheterojunction-based photoelectrochemical (PEC) biosensor to achieve the ultrasensitive detection of LncNR_040117. The nanoheterojunction aids in the effective separation of photogenerated carriers and significantly improve the photocurrent response of the biosensor. The conjugation of LncNR_040117 onto the PEC biosensing platform increased the steric hindrance between electrolyte and electrode, subsequently decreasing the photocurrent signal. The PEC biosensor showed a wide detection range of 0.1–10^6^ fM and a low limit of detection of 0.025 fM. For clinical sample testing, the results of the PEC and RT-qPCR were highly consistent. Overall, LncNR_040117 in PMPs was identified as an effective biomarker for RM/APS and could be accurately detected by the proposed PEC biosensor, which is expected to provide a reliable diagnostic platform for RM/APS.

**Supplementary Information:**

The online version contains supplementary material available at 10.1186/s12951-022-01608-1.

## Introduction

Recurrent miscarriage (RM) is an obstetric disease with a prevalence of 3% in women of childbearing age. The condition is associated with parental chromosomal anomalies, uterine abnormalities, endocrine factors, thrombophilia, cervical insufficiency and immunological disorders [1–6]. Of the various RM-related immunological disorders, antiphospholipid antibody syndrome (APS) is the most common [7, 8]. Previous studies have revealed that platelet-derived microparticles (PMPs), small vesicles that arise from platelets, play a crucial role in mediating immune disorders [9, 10]. The abnormal expression of long non-coding RNAs (LncRNAs) in PMPs is commonly linked to RM/APS, marking their potential as biomarkers for the condition [11, 12]. LncRNAs are a recently identified category of non-coding regulatory RNAs that participate in nearly all cellular activities investigated so far [13–15]. In literature, the dysfunction of LncRNAs is related to the occurrence and development of various diseases including RM/APS [16, 17]. Therefore, the identification and detection of RM/APS associated LncRNAs and understanding of their pathology requires attention.

In recent years, the application of a minimally to non-invasive form of detection technology, liquid biopsy, has flourished. This technique replaces the samples from tissue to blood or body fluids and requires only a trace amount of biological sample [18–20]. For isolated liquid containing dissolved nucleic acids, mature medical detection methods such as RT-qPCR, Northern blotting and microarray analysis have been clinically applied [21–23]. Nevertheless, they are limited by low sensitivity, high cost, poor portability and long waiting periods before detection. Therefore, it is in need of developing new detection methods. Recently, with the rapid development of new functional materials, especially nanomaterials, biosensing systems that utilize the special optical, electrical, magnetic, and interface properties of the materials have been explored extensively [24–27]. Nucleic acids biosensors based on fluorescence, colorimetry, SERS, SPR and electrochemical principles have shown satisfactory sensitivity and stability [28–32]. The photoelectrochemical (PEC) biosensor is a portable ultrasensitive detection device which is assembled based on the photoelectric conversion function of photosensitive materials [33, 34]. The presence of biomarkers (such as RNA) triggers changes in electrochemical signals that can be quantitatively analyzed, showing great potential in LncRNAs detection [35].

g-C_3_N_4_ is a two-dimensional semiconductor material with a graphite-like layered structure. Its stable physicochemical properties, simple synthesis and environmentally friendly characteristics promotes g-C_3_N_4_ as an excellent candidate in the field of photoelectric conversion [36, 37]. Despite its advantages, the wide band gap of 2.7 eV and rapid carrier recombination result in low light utilization efficiency of g-C_3_N_4_, limiting its application in photocatalysis and light-based sensing [38, 39]. Hence, modification of the material is necessary to overcome its shortcomings [40, 41]. For example, Chen et al*.* prepared sulfur-doped g-C_3_N_4_ by chemical modification for the electrochemical detection of methylated mercury [42]. The adjustment of g-C_3_N_4_ band gap improves its charge transfer efficiency and surface area resulting in to high sensitivity. β-In_2_S_3_ is another semiconductor holds a band gap of 2–2.3 eV, this feature in conjunction with its high carrier mobility has helped the material gain traction in pollutant degradation, water splitting and solar battery applications [43–46]. Nevertheless, pure β-In_2_S_3_ exhibits poor light utilization efficiency and photochemical stability attributed to the rapid recombination of carriers and photocorrosion that is inherent to metal sulfide semiconductors [47]. To improve the photoelectric performance of β-In_2_S_3_, breakthroughs have been made by adjusting its morphology, doping or constructing β-In_2_S_3_-based heterostructures [48, 49]. Taking the above-mentioned points into account, a β-In_2_S_3_@g-C_3_N_4_ nanoheterojunction-based PEC biosensor with improved light utilization efficiency and photochemical stability was generated.

**Scheme 1:**
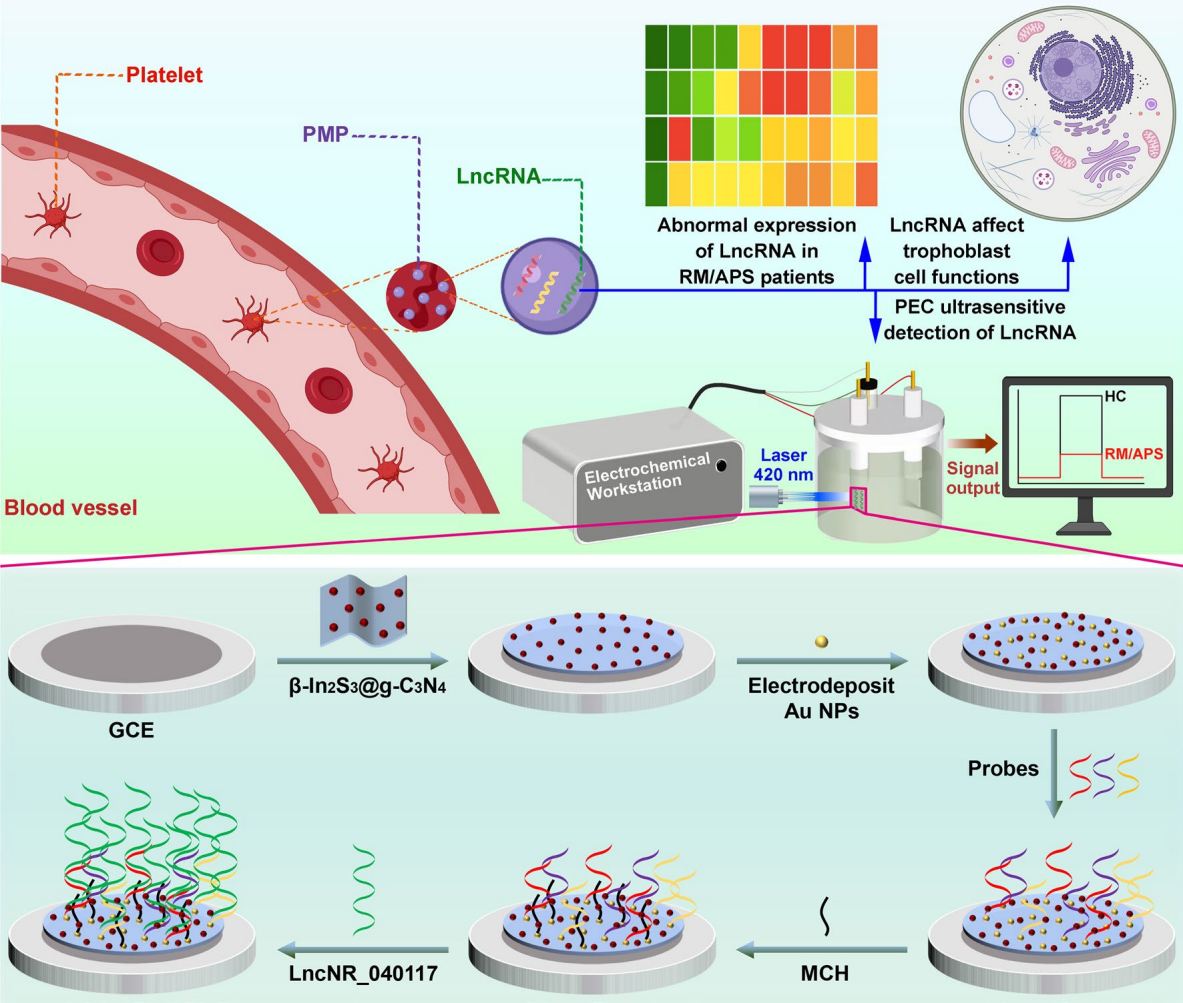
Schematic diagram of LncRNA identification and subsequent PEC detection of LncNR_040117

In this work, we identified LncNR_040117 in PMPs as the biomarker of RM/APS and successfully detected their presence through our proposed PEC biosensor. Scheme 1 illustrates the process for the identification of the function of LncNR_040117 and the fabrication of the β-In_2_S_3_@g-C_3_N_4_ nanoheterojunction-based PEC biosensor for ultrasensitive detection of LncNR_040117. PMPs of RM/APS patients and healthy controls were sorted by flow cytometry, and the expression of LncRNAs in PMPs was determined by microarray analysis and RT-qPCR. LncNR_040117 was proved to be highly expressed in RM/APS patients. The effect of downregulation of LncNR_040117 on the proliferation, migration, invasion, and apoptosis of trophoblast cells was studied to explore the correlation between LncNR_040117 and RM/APS. The regulatory effect of LncNR_040117 on the MAPK signaling pathway was also investigated to further confirm its relevance to RM/APS. The PEC biosensor based on β-In_2_S_3_@g-C_3_N_4_ nanoheterojunction exhibited excellent photoelectric conversion performance. A wide detection range of 0.1–10^6^ fM and a low calculated limit of detection of 0.025 fM for LncNR_040117 were obtained. The PEC biosensor distinguished LncNR_040117 from mismatch sequences and displayed excellent radiation stability. Furthermore, the PEC biosensor can exactly reflect LncNR_040117 concentrations in clinical samples, validating its feasibility for clinical application.

## Results and discussion

### Characterizations of PMPs and LncRNA expression profiles of PMPs

**Fig. 1:**
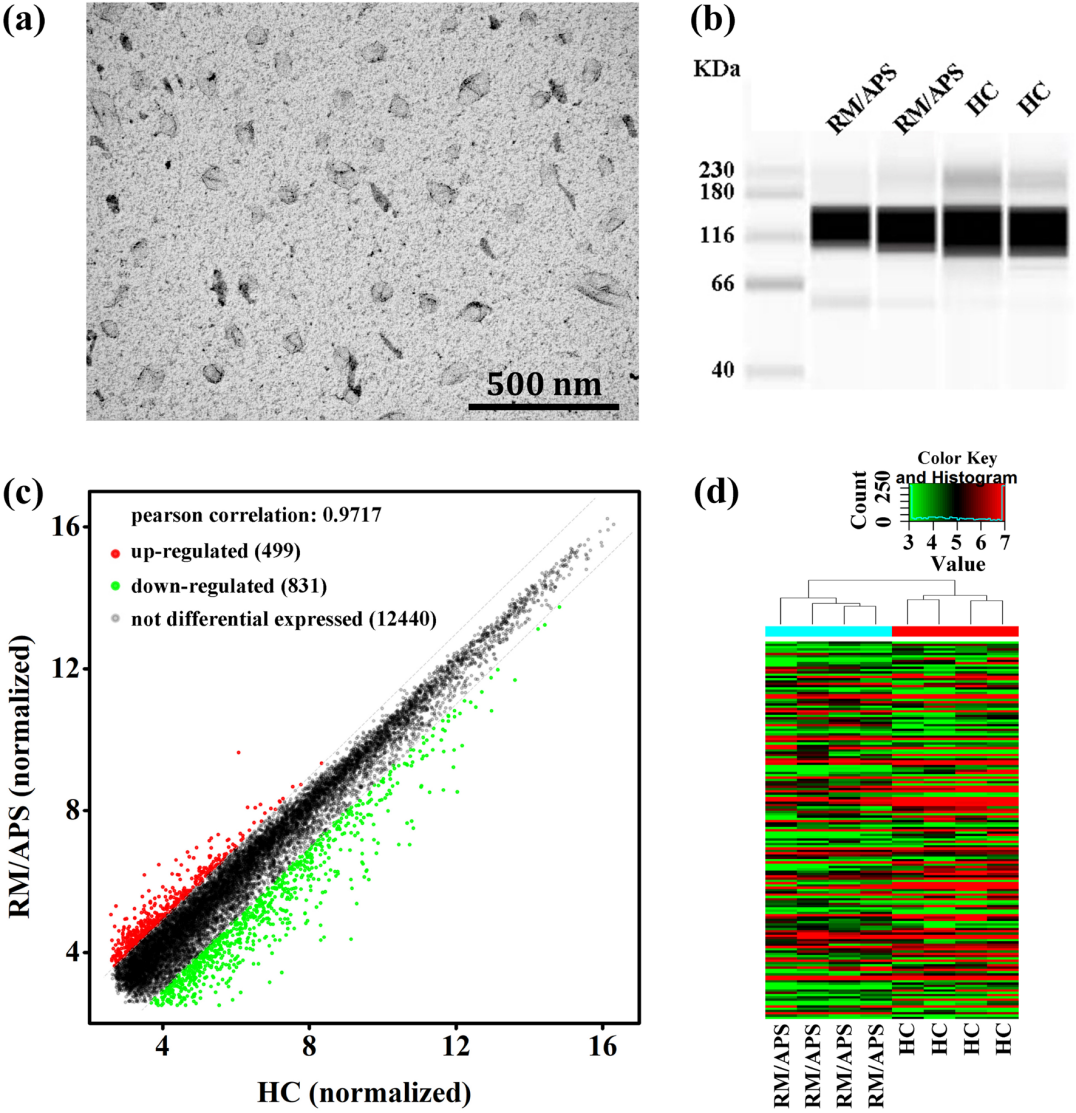
**a** TEM and **b** western blotting images of PMPs. **c** Volcano plot and **d** heatmap of LncRNAs expression

The TEM image of PMPs is shown in Fig. 1a. The PMPs were irregular fusiform to spherical in shape, and ranged from tens to hundreds of nanometers in size. The western blotting results (Fig. 1b) demonstrated that the concentration of CD41 protein, obtained from both RM/APS patients and healthy controls (HC) was high, indicative of the successful isolation of PMPs.

The LncRNA expression profile in PMPs, derived from RM/APS patients, was measured and visualized by the volcano plot and heatmap are presented in Fig. 1c and d, respectively. From LncRNA profiling, 1330 LncRNAs were shown to have significant differential expression levels in RM/APS patients compared to healthy controls during the 7–10 weeks gestational period, in which 499 LncRNAs were upregulated and 831 were downregulated, according to the cutoff criteria (P < 0.01 and |log2FC|> 2.0).

### LncNR_040117 as the biomarker of RM/APS

**Fig. 2:**
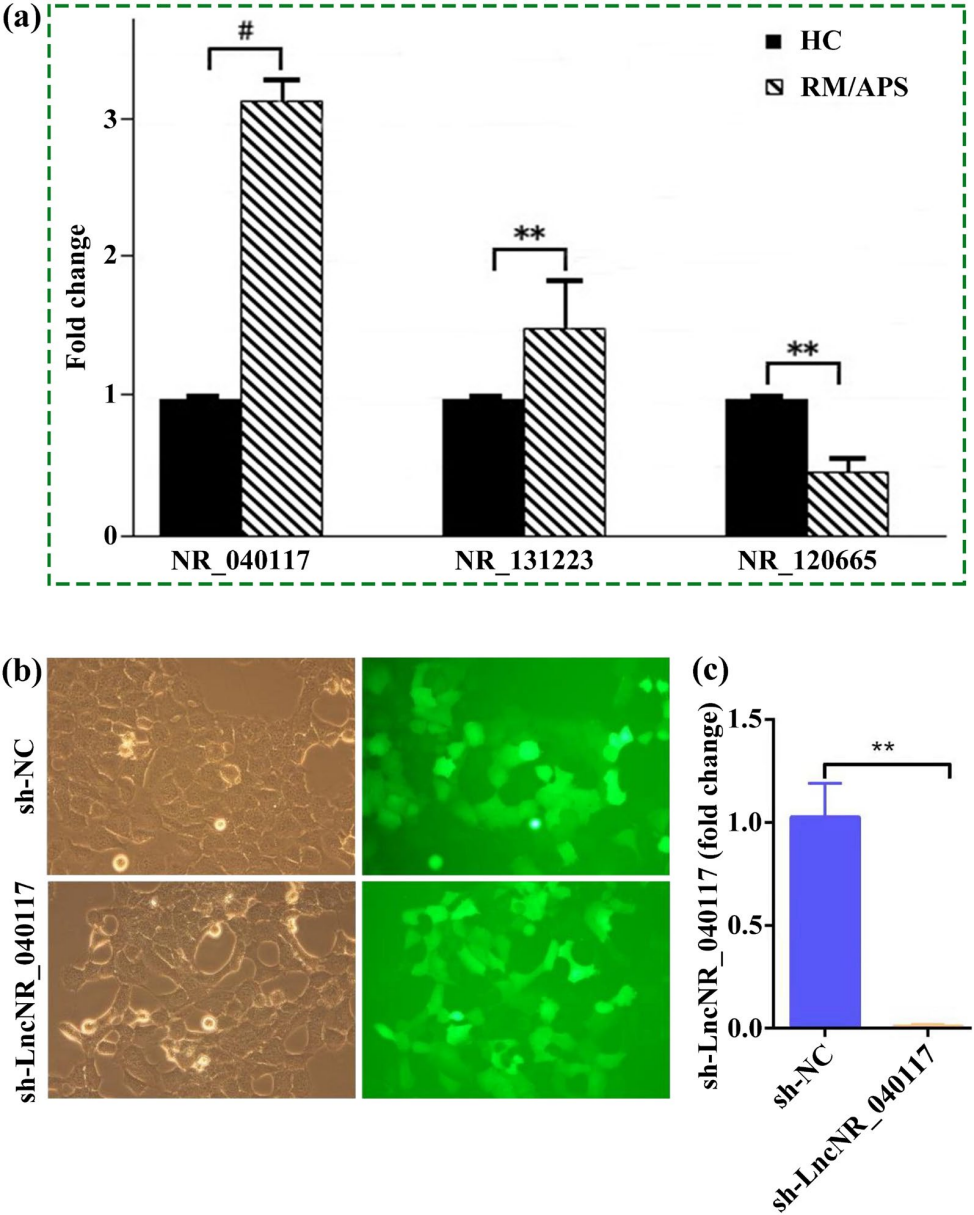
**a** Expression of LncNR_040117, LncNR_131223 and LncNR_120665 in PMPs, derived from RM/APS patients and compared to expression in healthy controls (n = 3, mean ± s.d.). **b** Light and fluorescence microscopy images of HTR-8/SVneo cells being or not being transfected by shRNA-NR_040117. (**c**) LncNR_040117 expression in HTR-8/SVneo cells being or not being transfected by shRNA-NR_040117 (n = 3, mean ± s.d.)

The abnormal expressions levels of LncNR_040117, LncNR_131223 and LncNR_120665 in PMPs from RM/APS patients were measured by the RT-qPCR method. As shown in Fig. 2a, the overexpression of LncNR_040117, LncNR_131223 and underexpression of LncNR_120665 were in consistent with the LncRNA profiling with their sequences listed in Additional file 1: Table S1. The difference in expression of LncNR_040117 between RM/APS patients and healthy controls was more significant compared to LncNR_131223 and LncNR_120665. Thus, LncNR_040117 and its potential as a RM/APS biomarker was selected for this study.

HTR-8/SVneo cells were transfected with shRNA-NR_040117 to downregulate the expression of LncNR_040117. The transfection rate exceeded 80% as indicated by the light and fluorescence microscopy images (Fig. 2b). The RT-qPCR results demonstrated that viability of the plasmids for LncNR_040117 knockdown. RT-qPCR analysis revealed that transfection with shRNA-LncNR_040117 reduced LncNR_040117 expression in HTR-8/SVneo cells than negative controls (Fig. 2c).

### Effect of LncNR_040117 downregulation on trophoblast cell functions and MAPK signaling pathway

**Fig. 3:**
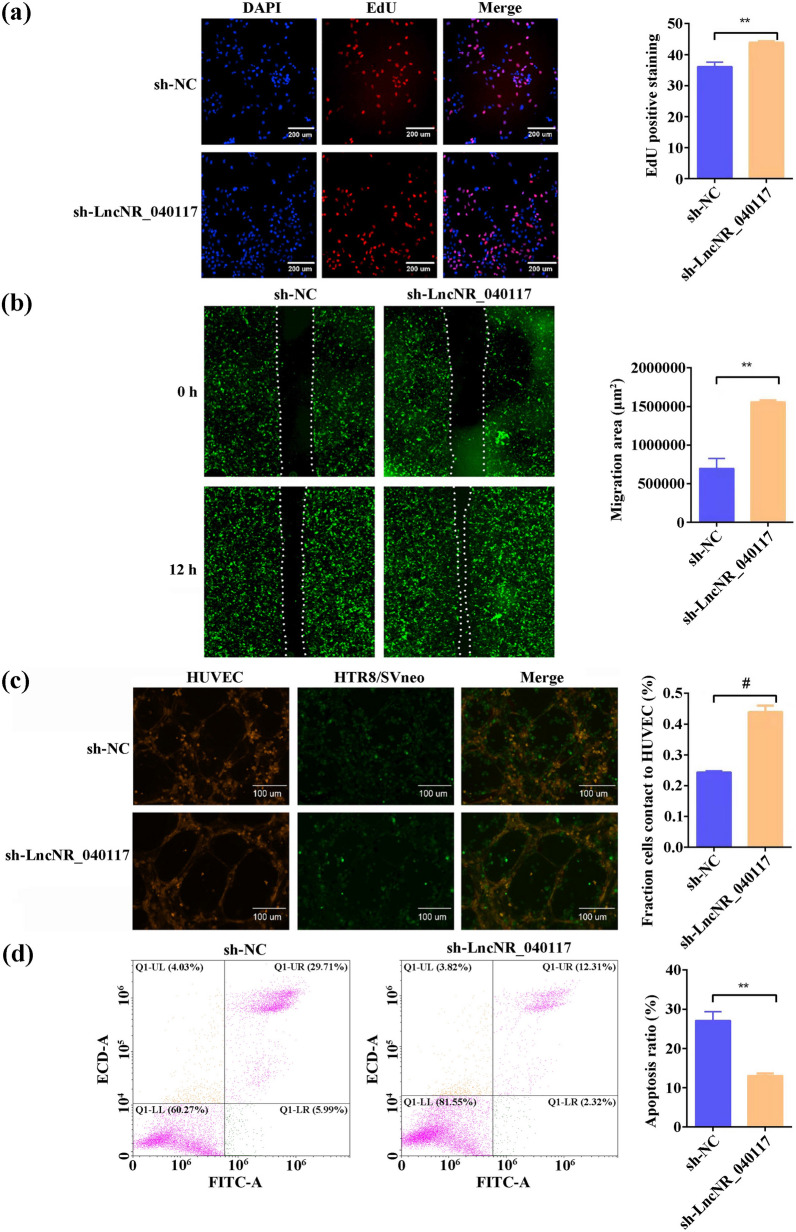
Effect of LncNR_040117 downregulation on trophoblast cell functions (n = 3, mean ± s.d.). **a** EdU assay, **b** scratch wound assay and **c** invasion assay of HTR8/SVneo cells before and after LncNR_040117 silencing. **d** Apoptosis of HTR8/SVneo cells measured by flow cytometry before and after LncNR_040117 silencing

The effect of LncNR_040117 downregulation on trophoblast cell functions was tested and the results are shown in Fig. 3. The EdU assay revealed that LncNR_040117 downregulation could increase the proliferative activity of HTR-8/SVneo cells than control cells (Fig. 3a). The migration and invasion of LncNR_040117 downregulation on HTR-8/SVneo cells were assessed through an in vitro migration assay and invasion assay, respectively. Downregulated LncNR_040117 could evidently facilitate migration, indicated by the higher wound closure rate compared to control cells (Fig. 3b). Invasion of trophoblasts was expressed by HTR-8/SVneo interactions with HUVEC. HTR-8/SVneo cells (green) were co-incubated with the established HUVEC tube network (red) for 6 h. Images were acquired with a 10 × objective and the percentage of HTR-8/SVneo cells in the tube is illustrated in Fig. 3c. The invasiveness of LncNR_040117 low-expression HTR-8/SVneo cells was significantly increased. Apoptosis rate of the two groups were assessed by FACS, as shown in Fig. 3d, where apoptosis rate was relatively lower in LncNR_040117 downregulation group. In summary, LncNR_040117 downregulation promoted the proliferation, migration, invasion and inhibited the apoptosis of trophoblast cells.

**Fig. 4:**
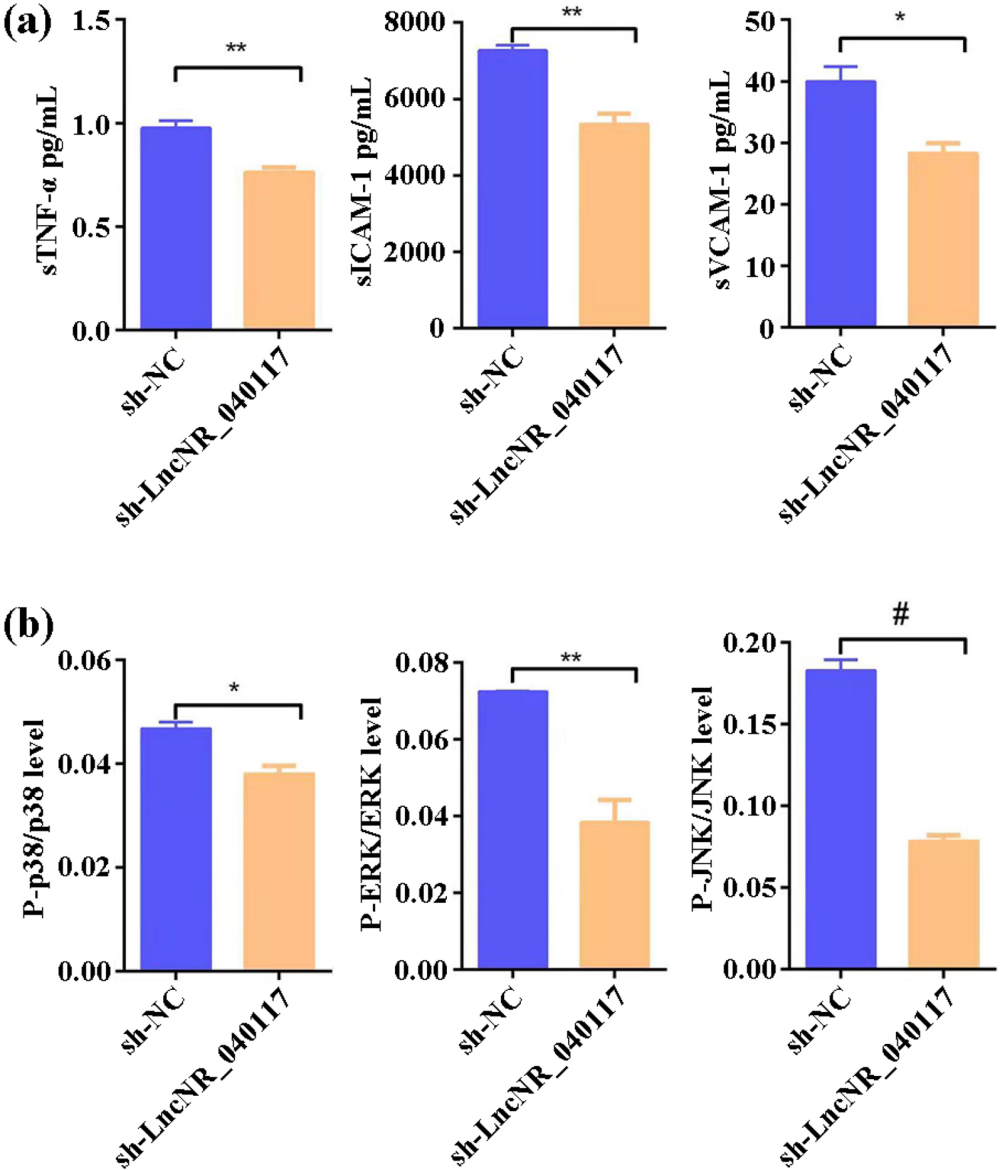
**a** The effect of LncNR_040117 on th protein expression of sTNF-α, sICAM-1 and sVCAM-1 (n = 3, mean ± s.d.). **b** The effect of LncNR_040117 on the levels of P-p38/p38, P-ERK/ERK and P-JNK/JNK (n = 3, mean ± s.d.)

Pervious study has revealed that LncRNAs act as activators by regulating the MAPK signaling pathway [50]. Based on this, the influence of LncNR_040117 downregulation on the expression of inflammatory factors (secreted TNF-α (sTNF-α), secreted ICAM-1(sICAM-1) and secreted VCAM-1 (sVCAM-1)) in addition to key molecules (P-p38/p38, P-ERK/ERK and P-JNK/JNK) of the MAPK signaling pathway were investigated. The data showed that LncNR_040117 was able to increase sTNF-α, sICAM-1 and sVCAM-1 protein expression (Fig. 4a), and the comparatively levels of P-p38/p38, P-ERK/ERK and P-JNK/JNK (Fig. 4b) suggested that LncNR_040117 activated MAPK signaling pathway. Hence, we may safely come to the conclusion that LncNR_040117 can act as an appropriate biomarker for RM/APS.

### Characterization of photosensitive materials

**Fig. 5:**
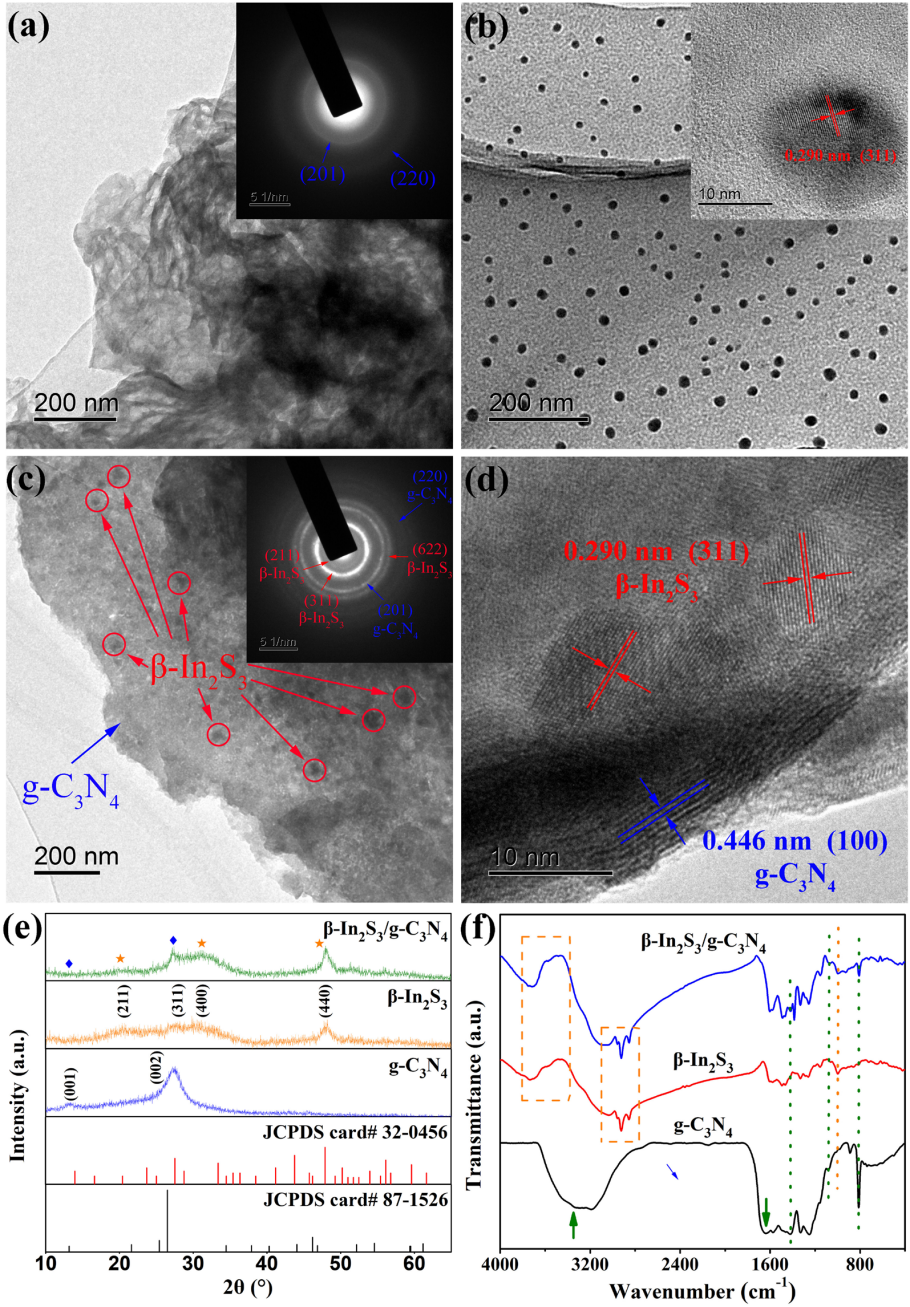
**a** TEM image and electron diffraction pattern of g-C_3_N_4_. **b** TEM images of β-In_2_S_3_ NPs. **c** TEM image and electron diffraction pattern of β-In_2_S_3_@g-C_3_N_4_ nanoheterojunction. **d** HRTEM image of β-In_2_S_3_@g-C_3_N_4_ nanoheterojunction. **e** XRD patterns and **f** FTIR spectra of g-C_3_N_4_, β-In_2_S_3_ NPs and β-In_2_S_3_@g-C_3_N_4_ nanoheterojunction

The TEM image and electron diffraction pattern of g-C_3_N_4_ are shown in Fig. 5a. g-C_3_N_4_ presented a two-dimensional layered morphology, corresponding to a large surface and a considerable number of reaction sites. The synthesis of g-C_3_N_4_ was proved by the electron diffraction pattern with diffraction rings attributed to (201) and (220) crystal planes. The TEM images of β-In_2_S_3_ NPs are shown in Fig. 5b. β-In_2_S_3_ exhibited an irregular spherical shape with a diameter of 9.4–23.7 nm. The lattice fringes, attributed to the (311) crystal plane of β-In_2_S_3_ NPs in the HRTEM image, confirmed successful synthesis of the NPs. The dense distribution of β-In_2_S_3_ NPs on g-C_3_N_4_ is displayed in Fig. 5c. The electron diffraction pattern confirmed the successfully preparation of the β-In_2_S_3_@g-C_3_N_4_ nanoheterojunction. Additional evidence includes the existence of the lattice fringes of (100) and (311) planes which were attributed to g-C_3_N_4_ and β-In_2_S_3_ respectively in the HRTEM image of β-In_2_S_3_@g-C_3_N_4_ nanoheterojunction (Fig. 5d).

The formation of β-In_2_S_3_@g-C_3_N_4_ nanoheterojunction was also proved by the XRD analysis, as displayed in Fig. 5e. XRD patterns of g-C_3_N_4_ and β-In_2_S_3_ NPs both showed broad diffraction peaks, indicative of their incomplete crystallization. The XRD pattern of the β-In_2_S_3_@g-C_3_N_4_ nanoheterojunction presented diffraction peaks attributed to the (001) and (002) crystal planes of g-C_3_N_4_ and (211), (400) and (440) crystal planes of β-In_2_S_3_, confirmation of the successful preparation of nanoheterojunction. FTIR analysis revealed the chemical composition and elemental bonding state of the β-In_2_S_3_@g-C_3_N_4_ nanoheterojunction. As shown in Fig. 5f, the bands attributed to the s-triazine ring and C−N stretching vibration of g-C_3_N_4_ at 810, 1073, and 1417 cm^−1^ appeared in the spectrum of β-In_2_S_3_@g-C_3_N_4_ nanoheterojunction, validating the existence of g-C_3_N_4_ [51]. Successful loading of β-In_2_S_3_ onto g-C_3_N_4_ was confirmed by the bands attributed to N−H, C−H and In − S stretching vibration of β-In_2_S_3_ at 3740, 3568, 2958, 2923, 2853 and 998 cm^−1^ in the spectrum of β-In_2_S_3_@g-C_3_N_4_ nanoheterojunction. β-In_2_S_3_@g-C_3_N_4_ nanoheterojunction exhibited the characteristic peaks attributed to g-C_3_N_4_ and β-In_2_S_3_, verifying the hybridization of g-C_3_N_4_ with β-In_2_S_3_. In addition, the peaks at 3340 and 1641 cm^−1^ attributed to the hydroxyl stretching and the vibration of g-C_3_N_4_ became weaker after the hybridization due to anhydrous reaction conditions.

The chemical state and elemental composition of β-In_2_S_3_@g-C_3_N_4_ nanoheterojunction was analyzed by XPS (Additional file 1: Fig. S1). The nanoheterojunction was composed of C, N, In, S and O elements (Additional file 1: Fig. S1a). The peak of O 1 s at 531.1 eV could be associated to that of surface-attached − OH [52]. The C 1 s spectrum was deconvoluted into three peaks (283.3, 284.8 and 286.7 eV), corresponding to C−H, C−C and N−C=N, respectively (Additional file 1: Fig. S1b). In the N 1 s spectrum (Additional file 1: Fig. S1c), three peaks at 397, 398 and 399.6 eV corresponding to the characteristic N − H, C = N − C and C − N − C in g-C_3_N_4_, respectively. For In 3d (Additional file 1: Fig. S1d) and S 2p (Additional file 1: Fig. S1e) spectra, the presence of In − S was clearly revealed. The β-In_2_S_3_@g-C_3_N_4_ nanoheterojunction exhibited the characteristic binding energy of both g-C_3_N_4_ and β-In_2_S_3_, further confirming the successful combination of g-C_3_N_4_ with β-In_2_S_3_.

### Photoelectric conversion mechanism of the PEC biosensing platform

**Fig. 6:**
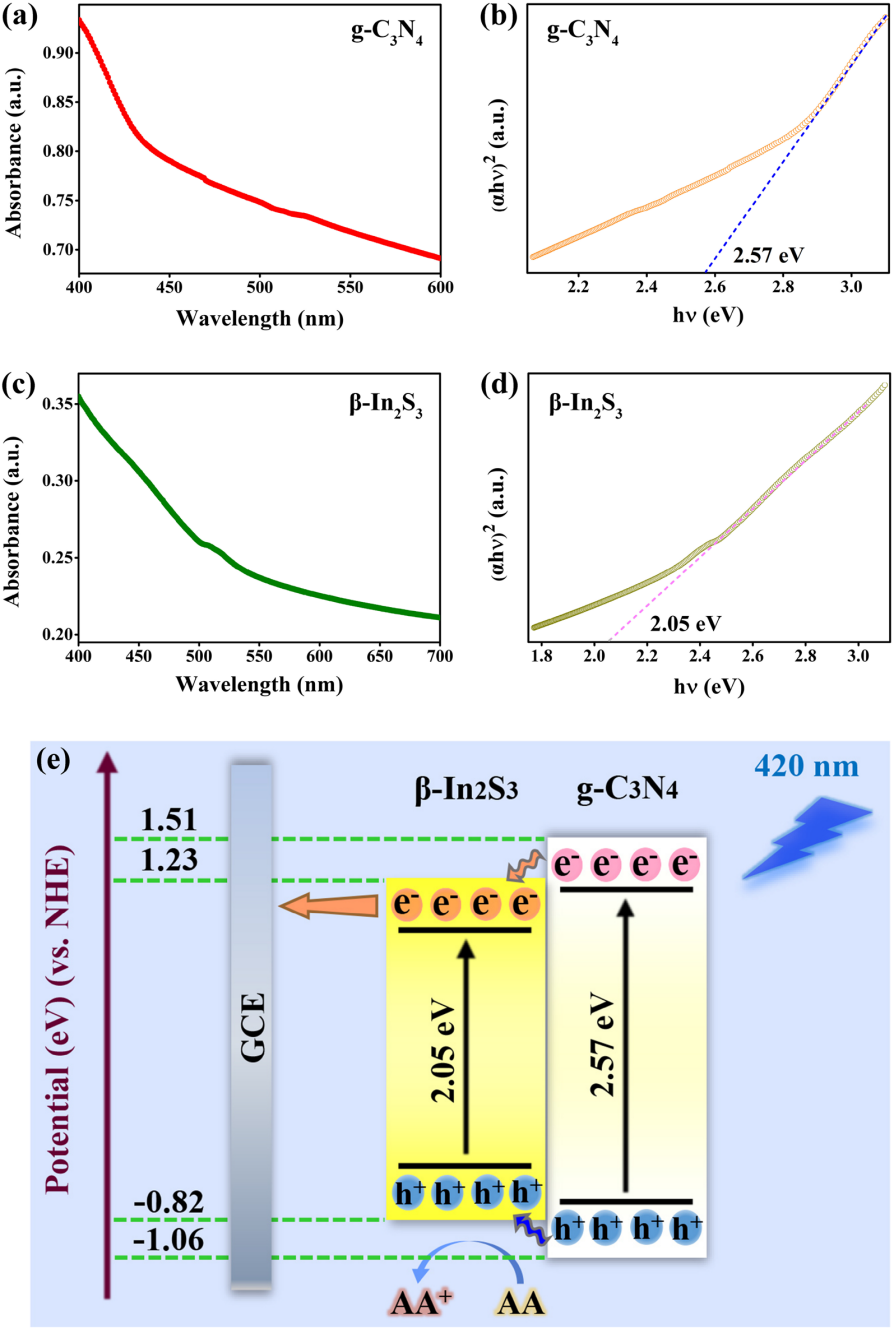
Absorption spectra as well as Tauc plots of **a**, **b** g-C_3_N_4_ and **c**, **d** β-In_2_S_3_ NPs. **e** Schematic diagram of β-In_2_S_3_@g-C_3_N_4_ nanoheterojunction band structure and working mechanism of PEC biosensing platform

The ultraviolet–visible light (UV–Vis) absorption spectra as well as Tauc plots of the g-C_3_N_4_ and β-In_2_S_3_ NPs are shown in Fig. 6a–d. From the absorption spectra, it was concluded that the g-C_3_N_4_ and β-In_2_S_3_ NPs can effectively absorb light with wavelengths shorter than 440 and 530 nm, respectively. Their band gaps (E_g_) were calculated according to the Tauc plot method and the corresponding Eq. 1 in the Additional file 1. The n value of g-C_3_N_4_ and β-In_2_S_3_ is 2 as they are direct bandgap semiconductors. Thus, as displayed in Fig. 6b and d, the E_g_ of g-C_3_N_4_ and β-In_2_S_3_ NPs were 2.57 and 2.05 eV, respectively, following Tauc plot method. The positions of conduction band (E_CB_) and valence band (E_VB_) were calculated according to the Eqs. 2–3 in the Additional file 1. The χ of g-C_3_N_4_ and β-In_2_S_3_ NPs were 4.73 and 4.71, respectively. The E_CB_ and E_VB_ of g-C_3_N_4_ were − 1.06 and 1.51 eV, respectively; while the E_CB_ and E_VB_ of β-In_2_S_3_ NPs were calculated as − 0.82 eV and 1.23 eV, respectively. The band structure and working mechanism of PEC biosensing platform is shown in Fig. 6e. β-In_2_S_3_@g-C_3_N_4_ was identified as type-I based on the band structures of g-C_3_N_4_ and β-In_2_S_3_. The photogenerated carriers of g-C_3_N_4_ could be transferred to β-In_2_S_3_, hindering the carrier recombination of g-C_3_N_4_. Subsequently, the electrons flow into the external circuit via GCE and the holes were reduced by ascorbic acid (AA) to form a circulating circuit.

### Signal response and analytical performance of the PEC biosensor

**Fig. 7:**
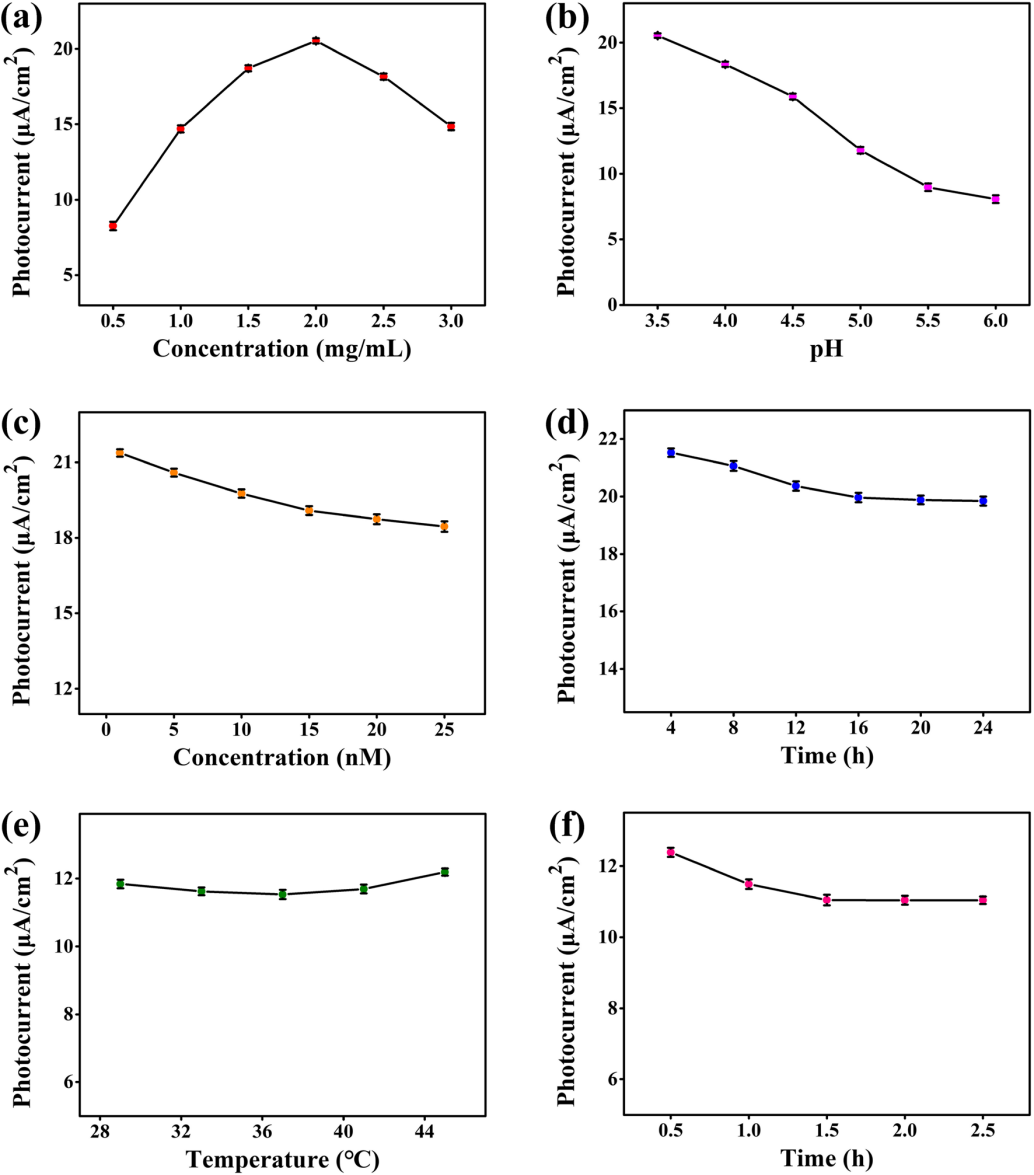
Effect of **a** β-In_2_S_3_@g-C_3_N_4_ nanoheterojunction concentration, **b** electrolyte pH, **c** probe concentration, **d** probe connection time, **e** hybridization temperature and **f** hybridization time on photocurrent response (the voltage was 0.08 V; n = 3, mean ± s.d.)

The measurement conditions including the concentration of In_2_S_3_@g-C_3_N_4_ nanoheterojunction, the pH of electrolyte, the concentration of probe, and the connection time of probe were optimized to ameliorate the detection performance of the PEC biosensor. The amount of photosensitizer has an opposing effect on the yield and transmission distance of photogenerated carriers [53]. As shown in Fig. 7a, a nanoheterojunction concentration of 2 mg/mL corresponded to the highest photocurrent. Hence, the optimal In_2_S_3_@g-C_3_N_4_ concentration of the nanoheterojunction was 2 mg/mL. The 0.01 M AA solution with an initial pH about 3.5 was regulated by adding MES buffer (0.5 M, pH 8.5). As shown in Fig. 7b, the photocurrent was observed to decrease with increasing pH. This may be due to the ions introduced by the MES buffer, hindering the reaction of photogenerated holes with AA. Hence, 0.01 M AA solution with a pH about 3.5 was selected as the optimal electrolyte. As shown in Fig. 7c, the poorly conductive probes reduced the photocurrent. Considering the target concentration is much lower than the probe and the photocurrent needs to be kept relatively high to observe a significant photocurrent change with the addition of the target, 10 nM was selected as the optimal probe concentration. The photocurrent was stable when the probe connection time is 16 h, as shown in Fig. 7d. This indicated that the probes were stably connected to the Au NPs for this duration. Thus, 16 h was the optimal connection time of probes.

**Fig. 8:**
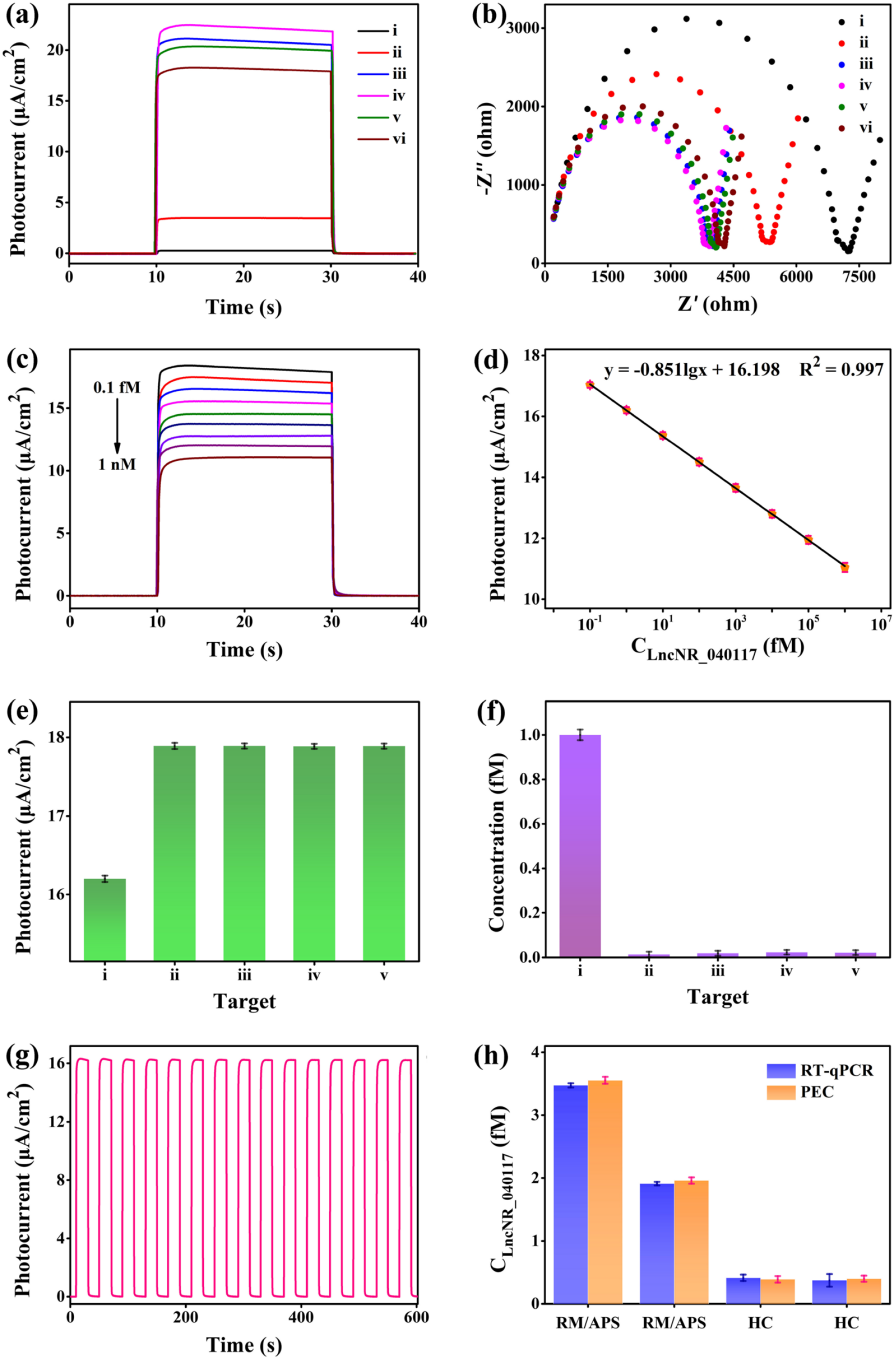
**a** Photocurrents and **b** EIS of the proposed PEC biosensor: (i) g-C_3_N_4_/GCE, (ii) β-In_2_S_3_/GCE, (iii) β-In_2_S_3_@g-C_3_N_4_/GCE, (iv) Au NPs/β-In_2_S_3_@g-C_3_N_4_/GCE, (v) probe/Au NPs/β-In_2_S_3_@g-C_3_N_4_/GCE, (vi) MCH/probe/Au NPs/β-In_2_S_3_@g-C_3_N_4_/GCE (the voltage was 0.08 V; the AC sine wave amplitude was 10 mV; the scan frequency range was 8 × 10^5^–10 Hz). **c** Photocurrent responses under different LncNR_040117 concentrations: 0, 0.1, 1, 10, 10^2^, 10^3^, 10^4^, 10^5^, 10^6^ fM. **d** Calibration line of photocurrent against the concentration of LncNR_040117 (n = 3, mean ± s.d.). **e** Photocurrent responses and **f** calculated concentrations of different sequences. Where i–v represent LncNR_040117, SNHG15 LncNR_152596.1, HOXA-AS2 LncNR_122069.1, RMRP LncNR_003051.3, and LUCAT1 LncNR_103548.1, respectively (n = 3, mean ± s.d.). **g** Photocurrent signal under 15 continuous radiation cycles at a LncNR_040117 concentration of 1 fM. **h** Detection results of LncNR_040117 concentrations in clinical serum samples by PEC biosensor and RT-qPCR method (n = 3, mean ± s.d.)

The photocurrent responses of different modified materials on the electrode surface are displayed in Fig. 8a. The coating of g-C_3_N_4_ showed a photocurrent of 0.26 μA/cm^2^, indicating its poor photoresponse activity. The coating of β-In_2_S_3_ displayed a photocurrent of 3.44 μA/cm^2^, significantly higher than g-C_3_N_4_, owing to its narrower band gap. The photocurrent of β-In_2_S_3_@g-C_3_N_4_ nanoheterojunction coating was 21.05 μA/cm^2^, significantly higher than the value of β-In_2_S_3_ and g-C_3_N_4_, demonstrating its efficient synergy and significantly enhanced photoelectric performance. The electrodeposition of Au NPs slightly increased the photocurrent to 22.34 μA/cm^2^ due to its impressive conductivity and surface plasmon resonance effect [54]. The connection of the thiol-DNA probes decreased the photocurrent to 20.19 μA/cm^2^. This is due to the poor conductivity of nucleic acids, subsequently increasing the steric hindrance between the electrolyte and electrode materials [55, 56]. The connection of the MCH further reduced the photocurrent to 18.15 μA/cm^2^, owing to its poor conductivity. In addition, the photocurrent response of GCE is displayed in Additional file 1: Fig. S2 and the value was lower than 0.015 μA/cm^2^. Meanwhile, the EIS measurement was performed to further understand the fabrication procedure of working electrode. Larger EIS semicircle radius represents larger charge transport resistance (R_CT_) of the electrolyte–electrode interface. As shown in Fig. 8b, the R_CT_ of curves i–vi were 6763, 4985, 3714, 3621, 3779 and 3962 Ω, respectively. The opposing photocurrent responses and R_CT_ trends of different modified materials verified the successful fabrication of the working electrode.

The variation of the photocurrent with changing LncNR_040117 concentrations was studied by incubating LncNR_040117 on the PEC biosensing platform. Before measuring the concentration of LncNR_040117, the probe-target hybridization temperature and time of were optimized. Figure 7e shows that the hybridization temperature of 37℃ corresponded to the lowest photocurrent under the constant hybridization time of 1 h. This is probably because lower temperatures led to slow hybridization rates while higher temperatures led to unstable hybrid duplexes [57]. Thus, 37℃ was considered to be the most suitable hybridization temperature. The photocurrent stabilized at 1.5 h at the hybridization temperature of 37℃, thus 1.5 h was determined to be the ideal optimal hybridization time (Fig. 7f) [58]. The photocurrent decreased with the increase of LncNR_040117 concentration due to the increase of steric hindrance (Fig. 8c). The fitted curve of photocurrent *vs.* LncNR_040117 concentration is shown in Fig. 8d. A good linear relationship in the LncNR_040117 concentration range of 0.1–10^6^ fM was showed. And the detection limit was 0.025 fM based on 3σ method. Compared with other PEC biosensors listed in Table 1, our fabricated PEC biosensor exhibited a broad range and a low limit of detection, demonstrating its potential for ultrasensitive detection of LncRNAs. The ultra-high sensitivity of the PEC biosensor was attributed to the excellent photoelectric conversion performance of the β-In_2_S_3_@g-C_3_N_4_ nanoheterojunction and the suitable design of the PEC biosensing platform.

**Table 1:** Comparison of PEC biosensors for RNA detection

Photosensitizer	Target	Linear range (fM)	Detection limit (fM)	Refs.
TiO_2_/Bi_2_S_3_	miRNA-141	10^3^–5 × 10^7^	200	[59]
WO_3_/Fe_2_O_3_	miRNA-21	10^2^–10^7^	36	[60]
Ti_3_C_2_:CdS	miRNA159c	10^2^–10^9^	33	[61]
CdTe-Bi_2_Te_3_	miRNA-21	10–10^5^	3.3	[62]
MB@NaKCN	miRNA-182-5p	10–10^8^	3.3	[63]
TiO_2_-CdS:Mn	miRNA-21	1–10^4^	0.5	[64]
CuO-CuWO_4_	miRNA-319a	1–10^5^	0.47	[65]
β-In_2_S_3_@g-C_3_N_4_	LncNR_040117	0.1–10^6^	0.025	This work
MoS_2_/AAO	miRNA-155	0.01–10^4^	0.003	[66]

The unmatched sequences of SNHG15 LncNR_152596.1, HOXA-AS2 LncNR_122069.1, RMRP LncNR_003051.3 and LUCAT1 LncNR_103548.1 with a uniform concentration of 1 fM were used as the controls to examine the detection selectivity. Figure 8e displayed the measured photocurrents. The photocurrent of LncNR_040117 was significantly smaller than other sequences. As shown in Fig. 8f, the calculated concentrations of SNHG15 LncNR_152596.1, HOXA-AS2 LncNR_122069.1, RMRP LncNR_003051.3 and LUCAT1 LncNR_103548.1 were approximately 0 according to the fitted line, demonstrating that this PEC biosensor specifically detects LncNR_040117. Irradiation stability is an important index to evaluate the availability of PEC biosensor. The photocurrent response of 15 continuous radiation cycles in Fig. 8g showed good repeatability with a relative standard deviation of 0.94%, proving the outstanding irradiation stability of the proposed biosensor.

The concentration of LncNR_040117 in clinical samples was measured to assess the reliability and practicality of PEC biosensor. The concentrations of LncNR_040117 in PMPs of two RM/APS patients and two healthy controls was measured by both RT-qPCR and the PEC biosensor. For RT-qPCR measurements, the concentration of LncNR_040117 was calculated according to the real-time fluorescence curves drawn by different concentrations of LncNR_040117. As shown in Fig. 8h, the concentration of LncNR_040117 in PMPs of RM/APS patients was found to be significantly higher than healthy controls. The values of C_PEC_/C_RT-qPCR_ of four clinical samples were reported 94.39–107.16%, demonstrating the impressive detection consistency of the two methods. The results demonstrated that this PEC biosensor can reliably detect LncNR_040117 concentration in clinical samples, showing the prospect of clinical diagnosis of RM/APS. Furthermore, due to the sequence modification flexibility of the probes, other LncRNAs can also be detected by this biosensor via simply changing the sequence of the probes.

## Conclusion

In summary, we identified LncNR_040117 in PMPs as a biomarker of RM/APS and realized its ultrasensitive detection by the fabricated β-In_2_S_3_@g-C_3_N_4_ nanoheterojunction-based PEC biosensor. LncNR_040117 in PMPs was found to upregulate in RM/APS patients through microarray analysis and RT-qPCR detection. LncNR_040117 downregulation increased the activity of HTR-8/SVneo cells and inhibited MAPK signaling pathway, demonstrating the biomarker potential of LncNR_040117 for RM/APS. The β-In_2_S_3_@g-C_3_N_4_ nanoheterojunction-based PEC biosensor was designed to achieve the ultrasensitive detection of LncNR_040117. The excellent photoelectric conversion effect of PEC biosensor was attributed to the formation of type-I heterostructure between β-In_2_S_3_ NPs and g-C_3_N_4_. The feasibility of ultrasensitive detection was attributed to the effective carrier separation, stable photosensitive materials, suitable PEC biosensing platform design, and optimal measurement conditions. The sensitivity, selectivity, stability, and accuracy of PEC biosensors for clinical samples detection were all satisfactory. To our knowledge, this is the first work for the ultrasensitive detection of LncRNAs by constructing a PEC biosensor. This work can serve a model for the identification and subsequent ultrasensitive detection of other LncRNA biomarkers, which is of great clinical application value.

## Materials and methods

The details of materials and methods are showed in the Additional file 1.

## Supplementary Information

**Additional file 1: Table S1.** Sequences of LncNR_040117, LncNR_131223 and LncNR_120665. **Figure S1.** XPS spectra of β-In_2_S_3_@g-C_3_N_4_ nanoheterojunction: (**a**) survey, (**b**) C 1s, (**c**) N 1s, (**d**) In 3d, and (**e**) S 2p. **Figure S2.** Photocurrent response of GCE.

## References

[1] Sato T, Migita O, Hata H, Okamoto A, Hata K (2019). Analysis of chromosome microstructures in products of conception associated with recurrent miscarriage. Reprod Biomed Online.

[2] Arab H, Alharbi AJ, Oraif A (2019). The role of progestogens in threatened and idiopathic recurrent miscarriage. Int J Womens Health.

[3] Meuleman T, Drabbels J, van Lith JMM (2018). Lower frequency of the HLA-G UTR-4 haplotype in women with unexplained recurrent miscarriage. J Reprod Immunol.

[4] Guerrero B, Hassouneh F, Delgado E, Casado JG, Tarazona R (2020). Natural killer cells in recurrent miscarriage: an overview. J Reprod Immunol.

[5] Kolben TM, Rogatsch E, Vattai A (2018). PPAR gamma expression is diminished in macrophages of recurrent miscarriage placentas. Int J Mol Sci.

[6] Lob S, Amann N, Kuhn C (2021). Interleukin-1 beta is significantly upregulated in the decidua of spontaneous and recurrent miscarriage placentas. J Reprod Immunol.

[7] Levy RA, dos Santos FC, de Jesus GR, de Jesus NR (2015). Antiphospholipid antibodies and antiphospholipid syndrome during pregnancy: diagnostic concepts. Front Immunol.

[8] Pelusa HF, Pezzarini E, Basiglio CL (2017). Antiphospholipid and antioangiogenic activity in females with recurrent miscarriage and antiphospholipid syndrome. Ann Clin Biochem.

[9] Zhou Q, Lian Y, Zhang Y (2019). Platelet-derived microparticles from recurrent miscarriage associated with antiphospholipid antibody syndrome influence behaviours of trophoblast and endothelial cells. Mol Hum Reprod.

[10] Kyselova A, Elgheznawy A, Wittig I (2020). Platelet-derived calpain cleaves the endothelial protease-activated receptor 1 to induce vascular inflammation in diabetes. Basic Res Cardiol.

[11] Wang Y, Liu HZ, Liu Y, Wang HJ, Pang WW, Zhang JJ (2019). Disordered p53-MALAT1 pathway is associated with recurrent miscarriage. Kaohsiung J Med Sci.

[12] Xie JY, Liang TT, Zhao JS (2021). Lnc-HZ08 regulates BPDE-induced trophoblast cell dysfunctions by promoting PI3K ubiquitin degradation and is associated with miscarriage. Cell Biol Toxicol..

[13] Kopp F (2019). Molecular functions and biological roles of long non-coding RNAs in human physiology and disease. J Gene Med.

[14] Omote N, Sauler M (2020). Non-coding RNAs as regulators of cellular senescence in idiopathic pulmonary fibrosis and chronic obstructive pulmonary disease. Front Med.

[15] Wang YY, Liu Q, Wang FC (2021). Potential roles of exosome non-coding RNAs in cancer chemoresistance. Oncol Rep.

[16] Zhu YC, Liu Q, Liao MJ (2019). Overexpression of lncRNA EPB41L4A-AS1 induces metabolic reprogramming in trophoblast cells and placenta tissue of miscarriage. Mol Ther-Nucl Acids.

[17] Che D, Yang YF, Xu YF (2019). The lncRNA MALAT1 rs619586 G variant confers decreased susceptibility to recurrent miscarriage. Front Physiol.

[18] Snow A, Chen D, Lang JE (2019). The current status of the clinical utility of liquid biopsies in cancer. Expert Rev Mol Diagn.

[19] Siravegna G, Marsoni S, Siena S, Bardelli A (2017). Integrating liquid biopsies into the management of cancer. Nat Rev Clin Oncol.

[20] Corcoran RB, Chabner BA (2018). Application of cell-free DNA analysis to cancer treatment. N Engl J Med.

[21] Hunt EA, Broyles D, Head T, Deo SK (2015). MicroRNA detection: current technology and research strategies. Annu Rev Anal Chem.

[22] Chey S, Claus C, Liebert UG (2010). Validation and application of normalization factors for gene expression studies in Rubella virus-infected cell lines with quantitative real-time PCR. J Cell Biochem.

[23] Mitrevska K, Milosavljevic V, Gagic M, Richtera L, Adam V (2021). 2D transition metal dichalcogenide nanomaterial-based miRNA biosensors. Appl Mater Today.

[24] Sun ZW, Yang JJ, Li H (2021). Progress in the research of nanomaterial-based exosome bioanalysis and exosome-based nanomaterials tumor therapy. Biomaterials.

[25] Ye YY, Xie MZ, Tang J, Ouyang JX (2019). Highly sensitive and tunable terahertz biosensor based on optical Tamm states in graphene-based Bragg reflector. Results Phys.

[26] Wang L, Lin JH (2020). Recent advances on magnetic nanobead based biosensors: from separation to detection. Trac Trends Anal Chem.

[27] Su S, Sun Q, Gu XD (2019). Two-dimensional nanomaterials for biosensing applications. Trac-Trends Anal Chem.

[28] Cheong J, Yu H, Lee CY (2020). Fast detection of SARS-CoV-2 RNA via the integration of plasmonic thermocycling and fluorescence detection in a portable device. Nat Biomed Eng.

[29] Tian YY, Zhang L, Wang LH (2020). DNA-functionalized plasmonic nanomaterials for optical biosensing. Biotechnol J.

[30] Ma XY, Guo ZZ, Mao ZQ, Tang YG, Miao P (2018). Colorimetric theophylline aggregation assay using an RNA aptamer and non-crosslinking gold nanoparticles. Microchim Acta.

[31] Muhammad M, Huang Q (2021). A review of aptamer-based SERS biosensors: design strategies and applications. Talanta.

[32] Peng Y, Pan YH, Sun ZW (2021). An electrochemical biosensor for sensitive analysis of the SARS-CoV-2 RNA. Biosens Bioelectron.

[33] Tu WW, Wang ZY, Dai ZH (2018). Selective photoelectrochemical architectures for biosensing: design, mechanism and responsibility. Trac Trends Anal Chem.

[34] Sun Z, Tong Y, Zhao L (1800). MoS_2_@Ti_3_C_2_ nanohybrid-based photoelectrochemical biosensor: a platform for ultrasensitive detection of cancer biomarker exosomal miRNA. Talanta.

[35] Wang MH, Yin HS, Zhou YL (2019). Photoelectrochemical biosensor for microRNA detection based on a MoS_2_/g-C_3_N_4_/black TiO_2_ heterojunction with Histostar@AuNPs for signal amplification. Biosens Bioelectron.

[36] Ong WJ, Tan LL, Ng YH, Yong ST, Chai SP (2016). Graphitic carbon nitride (g-C_3_N_4_)-based photocatalysts for artificial photosynthesis and environmental remediation: are we a step closer to achieving sustainability?. Chem Rev.

[37] Chen XJ, Shi R, Chen Q (2019). Three-dimensional porous g-C_3_N_4_ for highly efficient photocatalytic overall water splitting. Nano Energy.

[38] Zhang YW, Xu JS, Mei J (2020). Strongly interfacial-coupled 2D–2D TiO_2_/g-C_3_N_4_ heterostructure for enhanced visible-light induced synthesis and conversion. J Hazard Mater.

[39] Hu SS, Ouyang WJ, Guo LH (2017). Facile synthesis of Fe_3_O_4_/g-C_3_N_4_/HKUST-1 composites as a novel biosensor platform for ochratoxin A. Biosens Bioelectron.

[40] Zhao LL, Dong GH, Zhang L, Lu YF, Huang Y (2019). Photocatalytic nitrogen oxide removal activity improved step-by-step through serial multistep Cu modifications. ACS Appl Mater Interfaces.

[41] Vinh THT, Thi CM, Viet PV (2020). Enhancing photocatalysis of NO gas degradation over g-C_3_N_4_ modified alpha-Bi_2_O_3_ microrods composites under visible light. Mater Lett.

[42] Chen X, Sun X-T, Cui M-S, Liu Y, Cui K-P, Weerasooriya R (2021). Electrochemical determination of methylmercury via modulating bandgap of sulfur doped graphitic carbon nitride. J Environ Chem Eng.

[43] Zhang JJ, Wang H, Yuan XZ, Zeng GM, Tu WG, Wang SB (2019). Tailored indium sulfide-based materials for solar-energy conversion and utilization. J Photochem Photobiol C Photochem Rev.

[44] Pi YH, Jin S, Li XY, Tu S, Li Z, Xiao J (2019). Encapsulated MWCNT@MOF-derived In_2_S_3_ tubular heterostructures for boosted visible-light-driven degradation of tetracycline. Appl Catal B Environ.

[45] Sharma MD, Mahala C, Basu M (2020). Photoelectrochemical water splitting by In_2_S_3_/In_2_O_3_ composite nanopyramids. ACS Appl Nano Mater.

[46] Kumtepe A, Altaf CT, Sahsuvar S (2020). Indium sulfide based photoelectrodes for all-vanadium photoelectrochemical redox flow batteries. ACS Appl Energ Mater.

[47] Lee BR, Jang HW (2021). beta-In_2_S_3_ as water splitting photoanodes: promise and challenges. Electron Mater Lett.

[48] Xu R, Du Y, Liu L (2021). Molecular imprinted photoelectrochemical sensor for bisphenol A supported by flower-like AgBiS_2_/In_2_S_3_ matrix. Sens Actuator B Chem.

[49] Wang LJ, Zan L (2020). Facile one-pot solvothermal synthesis of noble metal-free NiS modified In_2_S_3_-based photocatalyst for highly efficient visible-light-driven Cr^6+^ removal. ChemistrySelect.

[50] Zhang YX, Yuan J, Gao ZM, Zhang ZG (2018). LncRNA TUC338 promotes invasion of lung cancer by activating MAPK pathway. Eur Rev Med Pharmacol Sci.

[51] Xing CS, Wu ZD, Jiang DL, Chen M (2014). Hydrothermal synthesis of In_2_S_3_/g-C_3_N_4_ heterojunctions with enhanced photocatalytic activity. J Colloid Interface Sci.

[52] Kokane SB, Sasikala R, Phase DM, Sartale SD (2017). In_2_S_3_ nanoparticles dispersed on g-C_3_N_4_ nanosheets: role of heterojunctions in photoinduced charge transfer and photoelectrochemical and photocatalytic performance. J Mater Sci.

[53] Pang XH, Zhang X, Gao KK (2019). Visible light-driven self-powered device based on a straddling nano-heterojunction and bio-application for the quantitation of exosomal RNA. ACS Nano.

[54] Peng B, Tang L, Zeng GM (2018). Self-powered photoelectrochemical aptasensor based on phosphorus doped porous ultrathin g-C_3_N_4_ nanosheets enhanced by surface plasmon resonance effect. Biosens Bioelectron.

[55] Meng H, Liu PK, Mo FJ, Chen M, Fu YZ (2020). A novel ultrasensitive photoelectrochemical biosensor for detecting microRNA 21 based on cosensitization strategy and p-n heterojunction quenching mode. Sens Actuator B Chem.

[56] Liu L, Zhu SY, Wei YM, Liu XL, Jiao SL, Yang JJ (2019). Ultrasensitive detection of miRNA-155 based on controlled fabrication of AuNPs@MoS_2_ nanostructures by atomic layer deposition. Biosens Bioelectron.

[57] Han YP, Zou R, Wang LY, Chen CY, Gong H, Cai CQ (2021). An amine-functionalized metal-organic framework and triple-helix molecular beacons as a sensing platform for miRNA ratiometric detection. Talanta.

[58] Zhang Y, Wang C, Zou XR, Tian XR, Hu J, Zhang CY (2021). Simultaneous enzyme-free detection of multiple long noncoding RNAs in cancer cells at single-molecule/particle level. Nano Lett.

[59] Zhao JG, Fu CP, Huang C (2021). Co_3_O_4_-Au polyhedron mimic peroxidase- and cascade enzyme-assisted cycling process-based photoelectrochemical biosensor for monitoring of miRNA-141. Chem Eng J.

[60] Sun JL, Li L, Ge SG (2021). Dual-mode aptasensor assembled by a WO_3_/Fe_2_O_3_ heterojunction for paper-based colorimetric prediction/photoelectrochemical multicomponent analysis. ACS Appl Mater Interfaces.

[61] Liu ST, Liu XP, Chen JS, Mao CJ, Jin BK (2020). Highly sensitive photoelectrochemical biosensor for microRNA159c detection based on a Ti_3_C_2_:CdS nanocomposite of breast cancer. Biosens Bioelectron.

[62] Yuan YL, Hu T, Zhong X, Zhu MH, Chai YQ, Yuan R (2020). Highly Sensitive photoelectrochemical biosensor based on quantum dots sensitizing Bi_2_Te_3_ nanosheets and dna-amplifying strategies. ACS Appl Mater Interfaces.

[63] Ding Q, Zhu MH, Deng HM, Yuan R, Yuan YL (2021). A novel self-enhanced carbon nitride platform coupled with highly effective dual-recycle strand displacement amplifying strategy for sensitive photoelectrochemical assay. Biosens Bioelectron.

[64] Wang B, Dong YX, Wang YL, Cao JT, Ma SH, Liu YM (2018). Quenching effect of exciton energy transfer from CdS: Mn to Au nanoparticles: a highly efficient photoelectrochemical strategy for microRNA-21 detection. Sens Actuator B Chem.

[65] Bingchen L, Huanshun Y, Yunlei Z, Minghui W, Jun W, Shiyun A (2018). Photoelectrochemical detection of miRNA-319a in rice leaf responding to phytohormones treatment based on CuO-CuWO_4_ and rolling circle amplification. Sens. Actuators B Chem..

[66] Jiao SL, Liu L, Wang JQ, Ma KJ, Lv J (2020). A novel biosensor based on molybdenum disulfide (MoS_2_) modified porous anodic aluminum oxide nanochannels for ultrasensitive microRNA-155 detection. Small.

