# Supplementary material for: Identification and ultrasensitive photoelectrochemical detection of LncNR_040117: a biomarker of recurrent miscarriage and antiphospholipid antibody syndrome in platelet-derived microparticles
Source: J Nanobiotechnology. 2022 Aug 31;20:396. doi: 10.1186/s12951-022-01608-1 (PMC9429728; doi:10.1186/s12951-022-01608-1)
Supplement: Supplementary file 1 — Additional file 1: Table S1. Sequences of LncNR_040117, LncNR_131223 and LncNR_120665. Figure S1. XPS spectra of β-In2S3@g-C3N4 nanoheterojunction: (a) survey, (b) C 1s, (c) N 1s, (d) In 3d, and (e) S 2p. Figure S2. Photocurrent response of GCE. [file 12951_2022_1608_MOESM1_ESM.docx]

**Supplementary Information**

**Identification and ultrasensitive photoelectrochemical detection of LncNR_040117: A biomarker of recurrent miscarriage and antiphospholipid antibody syndrome in platelet-derived microparticles**

Zhiwei Sun^1,†^, Qian Zhou^2,3,4,†^, Yufei Yang^1^, Lei Li^2,3,4^, Mengru Yu^2,3^, Hui Li^1^, Aihua Li^3^, Xietong Wang^2,3,4,^* and Yanyan Jiang^1,^*

^1^ Key Laboratory for Liquid−Solid Structural Evolution and Processing of Materials, Ministry of Education, Shandong University, Jinan, 250061, China.

^2^ Department of Obstetrics and Gynecology, Shandong Provincial Hospital Affiliated to Shandong First Medical University, Jinan, 250021, China.

^3^ Department of Obstetrics and Gynecology, Liaocheng People’s Hospital, Liaocheng, 252000, China.

^4^ Key Laboratory of Birth Regulation and Control Technology of National Health and Family Planning Commission of China, Maternal Child Health Hospital of Shandong Province, Jinan, 250014, China.

* Corresponding authors.

E-mail: wxt65@vip.163.com (X.T. Wang), [yanyan.jiang@sdu.edu.cn](mailto:yanyan.jiang@sdu.edu.cn) (Y.Y. Jiang).

^†^ These authors contributed equally to this work.

**Acquisition and sorting of clinical sample and characterizations of PMPs**

***Patients and samples***

All the recruited subjects are Chinese people from the Shandong Provincial Hospital. The RM/APS group consisted of 20 pregnant women at 7th-10th week gestation. The control group enrolled 20 healthy week-matched gestational women who have at least one child and no history of spontaneous miscarriage before the 10th gestational week. The blood samples were drawn from 9-11 am and were processed within 2 h. All participants provided written informed consent, and the specimen collection procedure was approved by the ethics committee of Shandong Provincial Hospital.

***Sorting PMPs by flow cytometry***

After centrifugation at 1500 g for 15 min and 13000 g for 2 min, platelet-free plasma (PFP) was obtained. 30 μL freshly thawed PFP was stained with 10 μL CD41-PE (Cat.# H20411-09H, Sungene Biotech) and 10 μL Annexin V-FITC (Cat.# AO2001-02H, Sungene Biotech). After 20 min, the mixture was diluted in 1 mL PBS. The PMPs sorting process was performed on a MoFlo cell sorter (Beckman Coulter).

***Characterizations of PMPs***

The morphology of PMPs was observed by a transmission electron microscope (TEM; JEM-1200EX, JEOL, Japan). The CD41 protein expression of PMPs from 2 RM/APS patients and 2 healthy controls was evaluated by a ProteinSimple Wes Capillary Western Blotting analyzer (ProteinSimple, USA). CD41 primary antibody (ab134131, Abcam) was diluted in an antibody diluent (1:50) and performed according to the standard manufacturer’s protocol of 12–230-kDa Jess separation module.

**LncRNA expression analysis and lentivirus silence shRNA transfection**

***Microarray analysis***

Microarray analysis was carried out by KangChen Biotechnology (Shanghai, China). The Arraystar Human LncRNA Microarray V4.0 used was designed for expression profiles of human LncRNAs. Approximately 40173 LncRNAs and 20730 coding transcripts can be detected by this fourth generation LncRNA microarray.

***Cell culture and lentivirus transfection***

All umbilical cords were obtained from the Department of Obstetrics and Gynecology of Shandong Provincial Hospital affiliated with Shandong First Medical University with written informed consent. The isolation method of HUVECs from umbilical cords was described in detail in our previous paper [1]. HUVECs were resuspended in complete endothelial cell medium containing 20 mg/mL endothelial cell growth supplement, 1% penicillin-streptomycin and 10% exosome-depleted FBS. All HTR-8/SVneo cells were cultured in DMEM/F12 containing 10% fetal bovine serum (FBS), penicillin (100 U/mL) and streptomycin (100 μg/mL) at 37˚C with 5% CO_2_. Silencing shRNA lentivirus transfection vector was purchased from the Genechem Co., Ltd. Cells were transfected when confluence reached 20%-30%. Speciﬁc knockdown was achieved by shRNAs against LncNR_040117 and nonspeciﬁc shRNA which were obtained from Genechem Co., Ltd. LncNR_040117 shRNA was designed to target the following sequence: (sh-LncNR_040117, sense: 5’-TCCAGCAAGGCAACATGGATA-3’; antisense: TATCCATGTTGCCTTGCTGGA; β-actin, sense: 5’-TTCCAGCAGATGTGGATCAGC-3’, antisense: 5’-GAAGCATTTGCGGTGGAC-3’). HTR-8/SVneo cells were seeded in 6-well plate and transfected with shRNA against LncNR_040117. Transfection with nonspeciﬁc shRNA served as a negative control (sh-NC). Following the manufacturer’s instructions, HTR-8/SVneo cells were transfected with 10 MOI lentivirus for 16 h. After the transfection, shRNA was removed and the cells were cultured for an additional 56 h. Transfection accuracy was evaluated by RT-qPCR as described in our previous work [1]. Cells were subcultured for cell proliferation, migration, invasion and apoptosis analysis.

**Trophoblast cell function analysis with LncNR_040117 downregulation**

***5-Ethynyl-2’-Deoxyuridine (EdU) assay***

3000 cells were seeded into 96-well plate and incubated for 24 h. The cells were cultured with 100 μL medium containing 50 μM EdU for 1h. Immunofluorescence staining was performed by an EdU reagent kit (C10310, Guangzhou RuiboBio Co., Ltd.). Images were obtained by high content screening (×10 objective). The positive incidence was proliferating cells/all cells observed and was defined as red dots/DAPI counts.

***Scratch wound assay***

A total of 10^4^ HTR-8/SVneo cells were seeded into 96-well plate and allowed to grow to 70% confluence in complete medium. The wound was scratched by a specific device made by Genechem Co., Ltd. and washed 3 times to remove cell debris. Cells were incubated for 12 h after scratching. Cells were observed to have migrated to the wound surface and the average length of the cells was photographed with high content screening (×4 objective).

***Invasion assay of interactions between HTR-8/SVneo cells and HUVECs***

A coculture system was used to determine the interactions of the HTR-8/SVneo cells and HUVECs. 24-well plates were covered with 150 μL Matrigel and incubated at 37 ℃ for 30 min, and then 1 × 10^5^ HUVECs were added to each well. Tube formation was monitored for the next 4 h, and then the formed tubes were dyed with DiI (red) for 1 h. HTR-8/SVneo cells were labeled PKH67 (green) for 10 min, and 1 × 10^5^ HTR-8/SVneo cells along with 1 × 10^7^ PMPs were seeded on the HUVEC tube structures. After 6 h of coculture, the cellular interactions were recorded with a Nikon Eclipse Ti Microscope (Nikon Instruments, Japan). Four images per well were photographed and quantified. The number of HTR-8/SVneo cells that were in contact with the HUVEC tubes/total number of HTR-8/SVneo cells was used to assess invasion ability of HTR-8/SVneo cells.

***Apoptosis analysis***

HTR-8/SVneo cells were seeded into 6-well plate and the apoptosis rate was detected by flow cytometer (CytoFLEX, Beckman Coulter). 10 μL Annexin-V and 5 μL PI were used to doubly stain apoptotic cells.

***Luminex xMAP***

Human cytokine MILLIPLEX^®^ MAP Kit (customized for sTNF-α, sICAM-1, sVCAM-1, P-p38/P38, P-ERK/ERK, P-JNK/JNK respectively) were purchased from Millipore-Sigma (Merck KGaA, Germany). A total of 10^4^ HTR-8/SVneo cells were seeded in a 96-well plate before centrifugation at 1500 g for 10 min and resuspended in 100 μL of DMEM/F12 without FBS followed by the addition of 10^6^ PMPs. The supernatant was collected and reduced to 50 μL after 48 h incubation. The levels of sTNF-α, sICAM-1, sVCAM-1, P-p38/P38, P-ERK/ERK, P-JNK/JNK were examined by a MILLIPLEX^®^ MAP kit on a Luminex 200. 25 μL original undiluted supernatant was incubated with magnetic beads overnight *via* shaking, and the plate was then washed three times. Detection antibodies were then added and incubated for 1 h at room temperature in the dark on a plate shaker. Streptavidin-phycoerythrin was added, followed by sheath fluid which was added three times after washing and served as the delivery medium to transport the sample to instrument optics. The concentrations were detected on the Luminex 200 with xPONENT software. Finally, the mean fluorescence intensity data was analyzed by Milliplex Analyst 5.1 software using a 5-parameter logistic model.

***Statistical analyses***

All the experiments were repeated independently at least 3 times and all the data analysis was performed on GraphPad Prism 5.0. One-way ANOVA was used to analyze the difference between 2 groups. P value < 0.05 was considered statistically significant (ns: P>0.05; ∗P<0.05; ∗∗P<0.01; #P<0.001).

**Preparation and characterization of photosensitizers**

***Raw materials for PEC biosensing***

Ultrapure water was used in all experiments. Urea (CH_4_N_2_O, AR grade), indium(Ⅲ) nitrate tetrahydrate (In(NO_3_)_3_·4H_2_O, metals basis), thioacetamide (C_2_H_5_NS, AR grade), octylamine (C_8_H_19_N, 99%), cyclohexane (C_6_H_12_, AR grade), tetrachloroauric acid (HAuCl_4_·3H_2_O, 99%), ascorbic acid (AA; C_6_H_8_O_6_, AR grade), 6-mercapto-1-hexanol (MCH; HS(CH_2_)_6_OH, 98%), MES buffer (0.5 M, pH8.5), and ethanol absolute (C_2_H_6_O, AR grade) were purchased from Shanghai Macklin Biochemical Co., Ltd. Sulfur was purchased from Sinopharm Chemical Reagent Co., Ltd. LncNR_040117, sulfhydryl modified DNA (SH-DNA) probes and unmatched sequences were purchased from Shanghai Biosune Biotechnology Co., Ltd. and their sequences are listed below.

LncNR_040117: AGGCTGTGTTTTCTCCCTCAGAACACGGGCACCCCACTGAGGGTCCTGCCTTCTGTGTTCTGGAGCCCCCCCTCAAGGAAGAAACCCGTGCTGTCTGCTCGCAACTCCAGGATGTTTGGACACCTCAGCCCCGTGAGGATCCCTCATCTCAGAGGCAAGTTTAACCTCAGACTTCCTTCATTAGATGAGCAGGTGATCCCAGCCAGGCTTCCGAAGATGGAGGTGAGGGCAGAAGAGCCCAAAGAAGCAACGGAGGTCAAAGACCAGGTAGAGACCCAGGAGCAGGAAGACAATAAAAGGGGCCCCTGTAGCAATGGGGAAGCAGCCTCCACCTCTAGGCCCCTGGAGACTCAGGGAAACCCCACTTCCCCCCGGTACAATCCCAGGCCCTTGGAGGGAAATGTCCAGCTCAAGAGCTTGACAGAAAACAACCAGACTGACAAGGCCCAGGTACATGCAGTGAGTTTCTACTCCAAGGGCCATGGAGTCGCCAGTTCACACAGCCCTGCTGGAGGCATTCTTCTCTTTGGGAAGCCTGACCCAGTTCCAACAGTGCTCCCTGCCCCAGTTCCAGGCTGCTCCCTGTGGCCAGAGAAGGCGGCCTTGAAGGTGCTGGGTAAAGACCACCTGCCCAGCTCTCCAGGTTTGCTGATGGTGGGGAAGGACATGCAGCCCAAGGATCCTGCAGCTCTTGGATCAAGTAGGTCTTCTCCACCCAGAGCTACCGGCTACAGGTCCCACAGGTCCCGCAAAAGAAAACTGTTGGGGCCACTGCCGCAGCTGCAACCAACCCCTCCCCTGCAACTGAGGTGGGATAGAGACGAGCCGCCCCCACCCGCTAAGCTTCCCTGCCTATCTCCTGAGGCACTGTTGGAGCTGGGTCAGGCTTCCCAAGGGGAAGGACGCCTCCAGCAAGGCAACATGGATAAGAACATGGGGATGTCCAAGAGATGAAAACAGCTGCTTGAGGGGAGAAAGAAGACATGGCAGGGCAGGTGTGGTGGCTCACGCCTG

SH-DNA probe 1:

5’SH-TCTAATGAAGGAAGTCTGAGG-3’

SH-DNA probe 2:

5’SH-CTTTTATTGTCTTCCTGCTCC-3’

SH-DNA probe 3:

5’SH-TTTCATCTCTTGGACATCCCC-3’

SNHG15 LncNR_152596.1:

GGGACTACGCGGTGACGTCGAGGTGCGCGGCGCAGCGCGCGGCGTCAGTCTTGGCTGGCAGACCTGTACTCCGTACTCCGTACTTCGTAGTCGCAGCGGCGCGGTCTTCGGCAGTCTAGTCATCCACCGCCATCCTGGGCCCCACGTGTTGCCTGACCATTCCTGAGCCCAGGTGGGAGCCGTGGCTGAGGTGACGGTCTCAAAGTGGAAGAGCTTACTGTCACAGCAACTCCTTTGCAAGATGCCCCGGTAAATCACGTTGCAGAGACCCCAACTCTGCCTTCCTGGAACTAGGCCTGCGTTTTCTCTGAACCATTATTTCATGGGGAAAATGAAATCTACTTCATGTGCCTGGTCTACTTACTCCTTCAGAAGTGATTTCTGAGCCGCCAGGAATAGTTGCTGAACACCCCAGGCCTGCTGAGGTCCCTCCTTGAGTCTCATGTTCAAGCAGTCTTTGTCCATGAAACTGGGAGGCGACCGTGTTAGCTGCCAGTTCCTGACAGCCACCTCTCACCAGTGGCTTCACTCTGTGTCCCTGACCCAGCACATGGCACAAGAGTGCCTGCCATCCGTCAGTGTTTCTACAGCAGCAATCCCAAGATGCTGGAGCTAGAGGGGACCTGACCTGAGAGAAGATACCTTCAGTGGCTGCCAGGCTGTTCCTTGGAACCTGTGCAGGGATGAGGCCTGCCTGTGTTAATACACCTAGTGAGGAGTGGAGCTGAATTTGAATGCAAGCCTTGGCACCTTAATTGAGCAAGTTTGAAACCTCGCTTGTTGCCCTTCTGGAAGGAGTCAGGAATTTCCAGTTCTGGGCCTGGGCTGTGGGTCTGGCAGACAGACCTCTGGCCCTAGGTTTGGGTGCCAGGTTCTCTGCTTCCAGAATGAGAAGCTTTGCTGTGCACCAGGACCTGGGCCCTTCTGGTATCTCCTGAATGAAAAACAAGGGATATTTAATAAATATGGATTTAAATATGTGA

HOXA-AS2 LncNR_122069.1:

GAAAAGGAAACGCCAAGACATAGAAAACCACGCTTTTCCCGTAGGAAGAACCGATGATGAGCCCTGATGAAAGAAGGAAGAAGACCCGCTGTCTGCGAAGGCCTAAAGGCCGCGGTTGCCAGGAACCGTGGAGGGCCAACTCCTCCCAACCGCCCTGGTGCAAAGTCCCACGCGGCGAAGAGTTTTGGAGCAGCGCTTACCTAGAAAGATGTTTAAATTCTGAACCAGGAATTGTCTCCAACTCCAGGCGCTCAGGGAATCGCCTTTTCCGGTGTCCAGGCGCTCTGCAGACAAATAAACAGCAGAAGCAAATGGTCACCGAGCCGGCAGTCAGCTTTCTGGGAGTGGGAGATGATGGGGAAAGAGGAAAGAATCGTCCGCTCGCCGGACCCTGGCTTGGAGAAGTTTGCGCTCCGCTGGGACTCTGCGGGCCCTTTGCGTCTACAGACCTATCCCTGCCCCGACTACCCCTTCACTCAGACCCAGGGAAGGACACGTTTCTATGCCTTACAGAGACTTGAAGCCTGAAAGCCTGGCCAAGTTTGGGAAGAAGAGGAGCCCTCTCAGAGCTCAAGAGCCTTCCTACTCTTTGGAACTTTTCCACAGTAGGCCAAGCTTGACAAGAGTTCAGCTCAAGTTGAACATACATACACACACTCTCACACACAAATTATGTGAGCCGTCAGAATCCAAGTGAATCCAGCTCAAGCTATCTACAAGGTTTTTACATGCAAGGTCAAGTATCTCAATCCAGAGGACTTTTGTTTTCTTAATGAAAAGCTTAGAAAACACATGAATCTTAGATTTTTAATGTTTTTTAAATGGAGTTTATTCTTAGCACATGGCTTTCTATGTAGCCACATCACAATTTGTACAGTTCCACATAAGTCTAAATGCACTCCCCTCTCCCCAAAGACCGTGCCCCAGAAGGGGACAACAGTATCTCTGTAACAGTGTCTTAAATAAATGCAAGTAAGAAAAACTAACATGTCACACCTACCATCAAGGTCTACACATCTTAAGAATTAAATAATCTTGTGAGGTCCA

RMRP LncNR_003051.3:

GGTTCGTGCTGAAGGCCTGTATCCTAGGCTACACACTGAGGACTCTGTTCCTCCCCTTTCCGCCTAGGGGAAAGTCCCCGGACCTCGGGCAGAGAGTGCCACGTGCATACGCACGTAGACATTCCCCGCTTCCCACTCCAAAGTCCGCCAAGAAGCGTATCCCGCTGAGCGGCGTGGCGCGGGGGCGTCATCCGTCAGCTCCCTCTAGTTACGCAGGCAGTGCGTGTCCGCGCACCAACCACACGGGGCTCATTCTCAGCGCGGCTGTAAAAAAAAA

LUCAT1 LncNR_103548.1:

AATCAACACTCCACTCAGACAATGCCCAGACCTCCAGAAACCATGTGTCAAGCTCGGATTGCCTTAGACAGGTGCAATTTAAGAACAGCTTTCATCCTCTTTTCTCTCATATTGTCACACTATGTGTTCTGACTTCTGGCTCCTTTCCTCACAAGAAGCTCACCCAGCTGGAACTCTTATGGGACCTTGGCACCAGAGACCACAAATTCCTCTTTGAAGTTTTCTAACAGCAACAATGGTATTTCTGACTTGGCTTTCTTGTATTTCTCTCACGTTAACAAAATTGGTTCAGCATCTACCATGGGCTACATGCTGAGCTACAGAGTTTCGCTCTGTCGCCCAGGCTGGAGTGCAGTGGCGCGCGATCTCGGTTCACTGCAAGCTCCACCTCCCGGGTTCACGCCATTCTCCTGCCTCAGCCTCCTGAGTAGCTGGGACTACAGGCGCCCGCCACCACACCCAGGAATCCAACTTGCTGTTTGCTATCACATGTGCTATACATGCTGTTGATGAAACTGCTAAAGGGGCTGAATGTGACTGACGTCTTTGGAAGGATGAGACTTAGCGTGCCTGTACAGTTGTGTCCAAATGCTGTCCTCATCTCCCAATGAAAAGGAACAAAACCCATCAGAAGATGTCAGAAGATAAGGATTTTTGTCCTGATGCTACACTTACCAGCTGTCCCTCAGTGTTCTACTTCTTAAAAAAAGAGAGATGGATAAACAGAGGCAACCCGAGGATAAAGGCCTTGCTCAGTGTCACACATTTCAGTCACTAAATAAGACACAATGGATGCCAGTATTCTCATCCCCTCACAAATAAAGAGCCTTCAAGCTCTTGCAGTCAACAAGAACTTTTGGAATGATTTCACTGCCTGAAAAGGCAGATAC

***Preparation of g-C_3_N_4_***

Urea (10 g) was added to the ceramic crucible and covered. The crucible was transferred to a muffle furnace and heated at 550℃ for 2 h. The g-C_3_N_4_ was obtained when the muffle furnace cooled to ambient temperature.

***Preparation of β-In_2_S_3_@g-C_3_N_4_ nanoheterojunction and β-In_2_S_3_ NPs***

The procedure for preparing the β-In_2_S_3_@g-C_3_N_4_ nanoheterojunction is as follows. Firstly, indium nitrate tetrahydrate (0.0764 g) and octylamine (5.0 mL) were added to a beaker containing cyclohexane (10.0 mL) and stirred for 10 min. Then, thioacetamide (0.0225 g), sulfur powder (0.007 g) and g-C_3_N_4_ (0.004 g) were added to the solution and stirred for 5 min. The mixture was then transferred to a Teflon-lined reactor and placed in a preheated oven at 160℃ for 4 h. The yellow product was washed repeatedly with absolute ethanol and dried *in vacuo* at 50℃ for 12 h. As a control, β-In_2_S_3_ NPs was also synthesized following the same procedure without the addition of g-C_3_N_4_ [2].

***Characterization of g-C_3_N_4_, β-In_2_S_3_ NPs and β-In_2_S_3_@g-C_3_N_4_ nanoheterojunction***

The morphology and structure of g-C_3_N_4_, β-In_2_S_3_ NPs and β-In_2_S_3_@g-C_3_N_4_ nanoheterojunction were observed by a high-resolution transmission electron microscope (HRTEM; JEM-2100, JEOL). The X-ray diffraction patterns of the g-C_3_N_4_, β-In_2_S_3_ NPs and β-In_2_S_3_@g-C_3_N_4_ nanoheterojunction were collected by an X-ray diffractometer (XRD; DMAX-2500PC, Rigaku). The Fourier transform infrared spectra (FTIR) of g-C_3_N_4_, β-In_2_S_3_ NPs and β-In_2_S_3_@g-C_3_N_4_ nanoheterojunction were collected by a Fourier transform infrared spectrometer (Tensor 37, Bruker) from 4000 to 400 cm^−1^. The X-ray photoelectron spectroscopy (XPS) spectrum of the β-In_2_S_3_@g-C_3_N_4_ nanoheterojunction was recorded on an AXIS Supra spectrometer (Shimadzu) using monochromatized Al Kα excitation. The ultraviolet-visible (UV-Vis) absorption spectra of g-C_3_N_4_ and β-In_2_S_3_ NPs were collected by an UV-Vis spectrophotometer (Specord 200 plus, Analytikjena).

**Fabrication of PEC biosensing platform and detection of LncNR_040117**

***Fabrication of PEC biosensing platform***

The schematic diagram of the PEC biosensing platform is shown in **Figure 1**. Firstly, In_2_S_3_@g-C_3_N_4_ nanoheterojunction aqueous solution (10 μL, 2 mg/mL) was dropped on glassy carbon electrode (GCE, diameter 3 mm) and dried at 25℃ for 4 h. Then, the Au nanoparticles (Au NPs) were electrodeposited on the surface of In_2_S_3_@g-C_3_N_4_ nanoheterojunction at -0.2 V for 30 s with 0.03 M HAuCl_4_ as the electrolyte. Subsequently, SH-DNA probe solution (10 μL, 10 nM) was dropped on the surface and reacted at 4℃ for 16 h to bind the probe through the Au-S bond. The electrode was washed with ultrapure water to remove the physically adsorbed probe. Finally, MCH (10 μL, 0.1 μM) solution was dropped and reacted for 30 min to block non-specific adsorption sites. Physically adsorbed MCH was removed by washing with ultrapure water.

***PEC detection of LncNR_040117***

Firstly, the target (10 μL) was dropped on the PEC biosensing platform and incubated at 37℃ for 1.5 h. The electrode was washed by ultrapure water to remove the unattached target. PEC detection and EIS measurements were carried out with a three-electrode system (DH7000, Jiangsu Donghua Analysis Instruments Co., Ltd, China). The platinum sheet (2 × 2 cm) and Ag/AgCl electrode were used as the counter electrode and reference electrode, respectively. The AA aqueous solution (0.01 M), saturated with N_2_, was used as the electrolyte. An LED lamp with an emission wavelength of 420 nm and an output power of 170 W was used to illuminate the working electrode. The measurement initiated after the electrode was immersed in the electrolyte and was retained in solution for 2 min to ensure that the system stability. The voltage for chronoamperometry was set to 0.08 V. The radiation light was turned on at 10 s and turned off at 30 s. For electrochemical impedance spectroscopy (EIS) measurements, an AC sine wave with 10 mV amplitude was utilized to disturb the steady-state open circuit voltage. The scan frequency range was 8 × 10^5^–10 Hz. All measurements were parallel performed for three times and the averages were reported.

**Equation for calculating the band gap**

(αhν)^n^ = B(hν - E_g_) (1)

Where α is the absorbance; h is the absorbance constant; ν is the light frequency; n is 2 for direct band gap semiconductors and 0.5 for indirect band gap semiconductors; B is a constant.

**Equations for calculating the E_VB_ and E_CB_**

E_CB_ = χ – E_c_ – 0.5E_g_ (2)

E_CB_ = E_VB_ - E_g_ (3)

Where χ is the geometric mean of the absolute electronegativity of each atom of the semiconductor. E_c_ equal to 4.5 eV is a constant relative to the normal hydrogen electrode (NHE).

**Table S1** Sequences of LncNR_040117, LncNR_131223 and LncNR_120665.

| **LncRNAs** | **Sequences from 5’ to 3’** |
| --- | --- |
| LncNR_040117 | Forward：TACCGGCTACAGGTCCCACA  Reverse：TGACCCAGCTCCAACAGTG |
| LncNR_131223 | Forward：CGGCTCTGGAAGCTAGAGG  Reverse：ATGTGGTGGCTGGTGGTCAAC |
| LncNR_120665 | Forward：GTTTCTGGGGACGCGAAGAT  Reverse：TGTTCAGCATCCCTGGCTAAG |


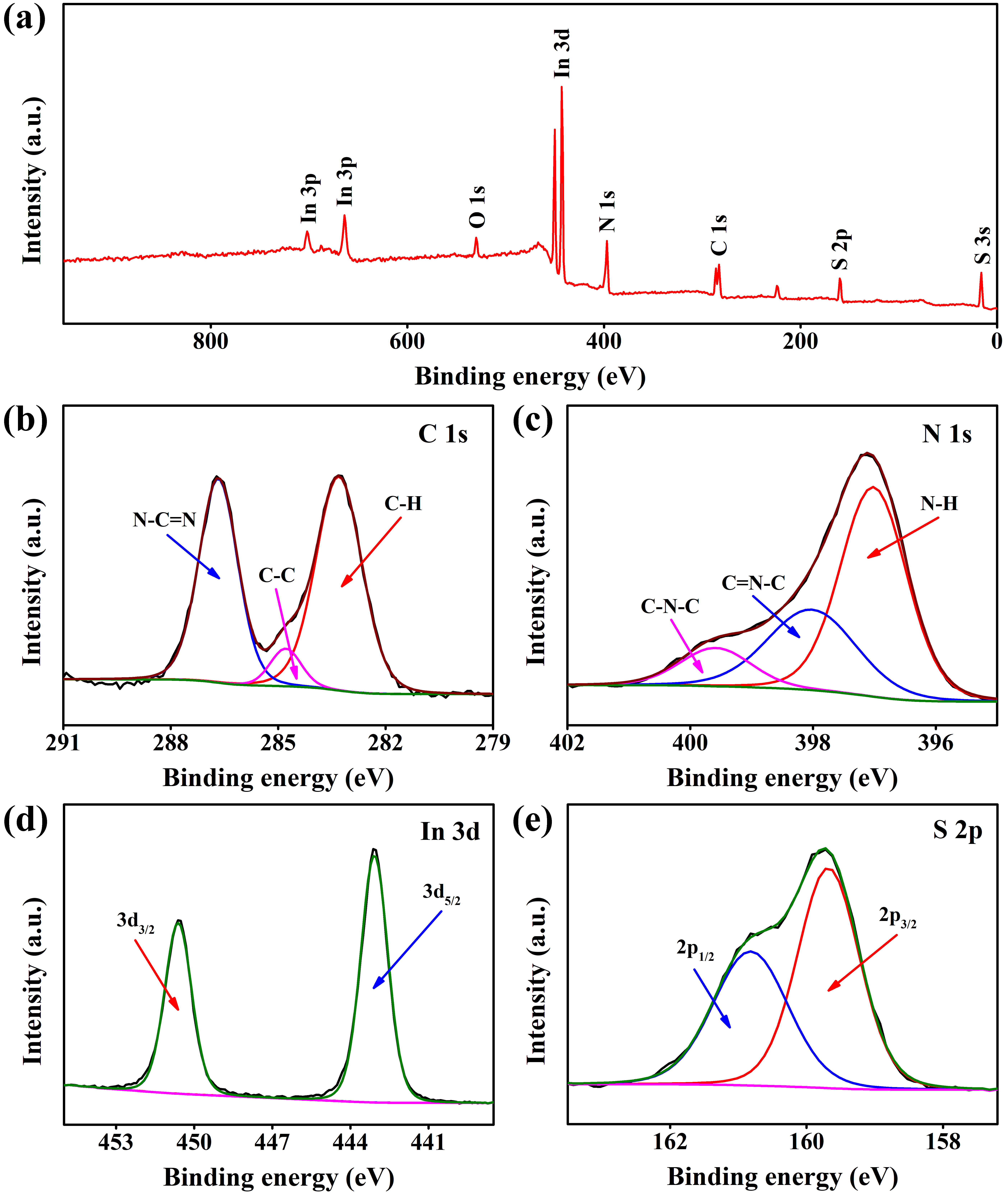


**Fig. S1** XPS spectra of β-In_2_S_3_@g-C_3_N_4_ nanoheterojunction: (**a**) survey, (**b**) C 1s, (**c**) N 1s, (**d**) In 3d, and (**e**) S 2p.


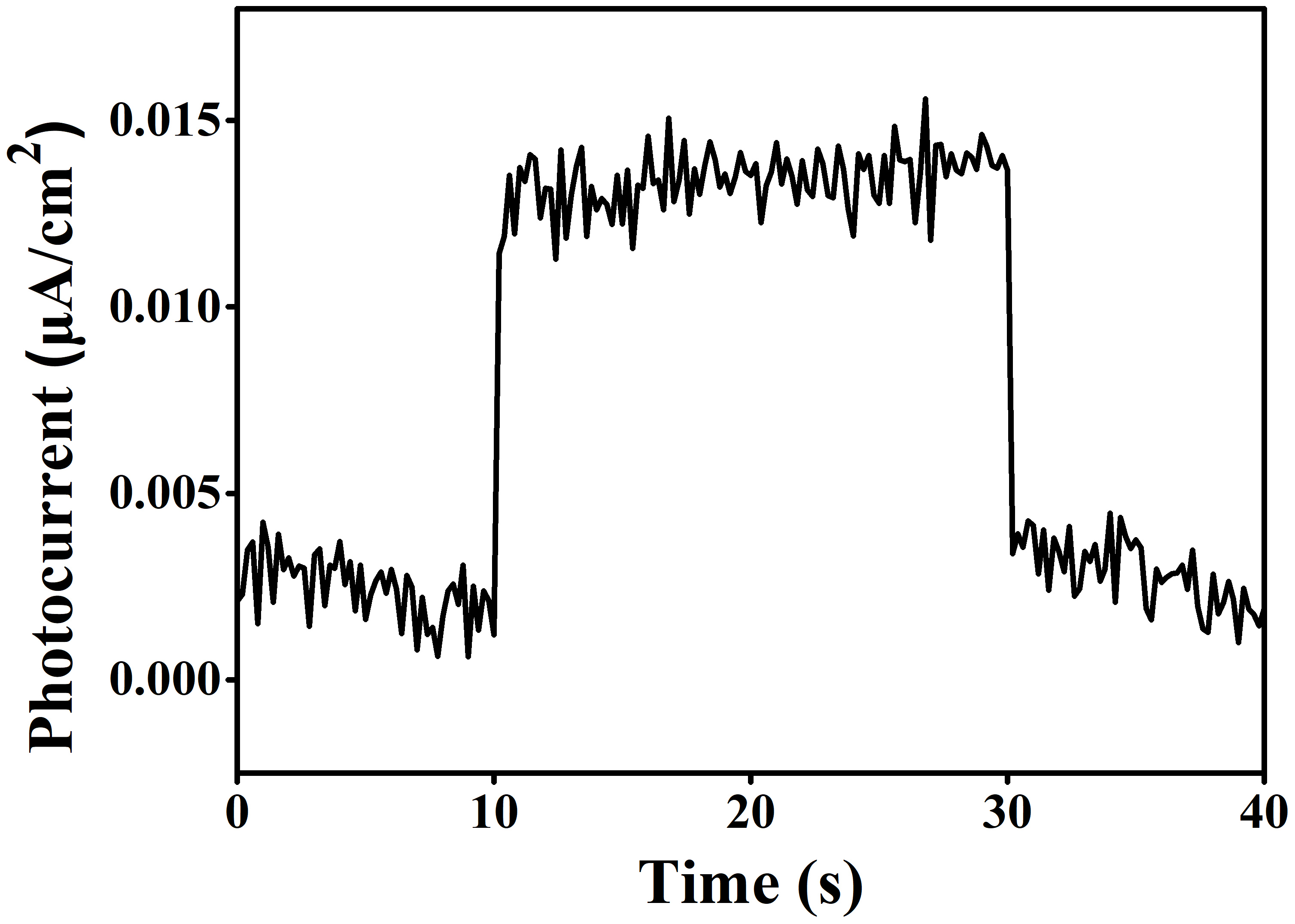


**Fig. S2** Photocurrent response of GCE.

**References**

1. Zhou Q, Lian Y, Zhang Y, et al. Platelet-derived microparticles from recurrent miscarriage associated with antiphospholipid antibody syndrome influence behaviours of trophoblast and endothelial cells. Mol. Hum. Reprod. 2019;25(8):483-94.

2. Tian Y, Wang LG, Tang HQ, Zhou WW. Ultrathin two-dimensional beta-In_2_S_3_ nanocrystals: oriented-attachment growth controlled by metal ions and photoelectrochemical properties. J. Mater. Chem. A. 2015;3(21):11294-301.
